# Decoding Triphenotypic Neutrophils in Cervical Cancer Evolution and Targeting SPP1+/GBP1+/ELOVL5+ Tumor‐Associated Neutrophils to Sensitize Immunotherapy

**DOI:** 10.1002/advs.202515511

**Published:** 2025-11-28

**Authors:** Xingyu Chang, Xinyu Qu, Tingting Ren, Tong Wu, Weiyong Gu, Jingxin Ding, Keqin Hua, Junjun Qiu

**Affiliations:** ^1^ Obstetrics & Gynecology Hospital of Fudan University, Shanghai Key Lab of Reproduction and Development Shanghai Key Lab of Female Reproductive Endocrine Related Diseases Shanghai 200433 China

**Keywords:** cervical cancer, immunotherapy, neutrophil, tumor immune microenvironment

## Abstract

Enhancing cervical cancer (CC) immunotherapy requires deciphering the heterogeneous tumor immune microenvironment (TIME), particularly neutrophil phenotypic dynamics. Here, 1) we collected 543 CC cases to find that patients with elevated neutrophil levels have a higher incidence of high‐risk pathological factors. 2) Three tissue‐specific neutrophils stages are revealed across cervical carcinogenesis. Specifically, in the normal stage, normal‐associated neutrophils (NANs) served as defenders to predominantly exert immune surveillance. In the HSIL stage, precancerous‐associated neutrophils (PANs) served as agitators to boost inflammation‐cancer transition. 3) In the CC stage, tumor cells and tumor‐associated neutrophils (TANs) engage in a detrimental “positive feedback loop” that drives CC aggressiveness. Specifically, tumor cells educated TANs to overexpress SPP1, GBP1, and ELOVL5, which subsequently activated three key mechanisms: promoting angiogenesis (SPP1‐NF‐κB‐HIF/VEGF), immunosuppression (GBP1‐NF‐κB‐PD‐L1), and dysregulated oxidative lipid metabolism (ELOVL5‐NF‐κB), leading to poor prognosis. 4) Finally, to explore the therapeutic value of TANs, we performed in vivo experiments, demonstrating that the combination of anti‐Ly6G and anti‐PD1 therapy resulted in improved anti‐tumor efficacy compared with anti‐PD1 monotherapy for CC. In conclusion, the study innovatively elucidated the three tissue‐specific neutrophils stages in the progression of “normal‐HSIL‐CC”, providing novel insights into TANs as potential targets for improving CC immunotherapeutic efficacy.

## Graphical abstract

The functional schematic diagram of tumor associated neutrophils.

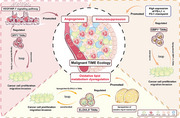

## 1 Introduction

Cervical cancer (CC) is a prevalent malignant tumor that ranks fourth in terms of incidence among all cancers and is a leading cause of cancer‐related mortality among women.^[^ ^1^ ^]^ In recent years, with advancements in precision medicine, immunotherapy has emerged as a promising treatment option for patients with CC. However, the relatively low overall response rate observed with immunotherapy in CC is a pressing concern.^[^ ^2^ ^]^ Extensive research has emphasised that the limited efficacy of CC immunotherapy is mainly attributed to the highly heterogeneous tumor immune microenvironment (TIME) composed of diverse cell types.^[^ ^3^, ^4^ ^]^ Therefore, it is crucial to elucidate the cellular characteristics within the TIME of CC to pave the way for improving the efficacy of immunotherapy and identifying novel therapeutic targets.

Among the numerous cell types that make up the TIME, myeloid cells are receiving increasing amounts of interest.^[^ ^5^, ^6^ ^]^ Nevertheless, the significance of granulocytes, particularly neutrophils, has been overlooked due to both technological constraints and their inherent fragility and short lifespans.^[^ ^7^ ^]^ Fortunately, with breakthroughs in high‐throughput sequencing technologies, the significance of neutrophils in tumor progression is gradually being more highly valued.^[^ ^8^ ^]^ The conventional perception of neutrophils as passive bystanders in the tumor immune response is now being challenged.^[^ ^9^ ^]^ Instead, neutrophils represent a remarkably plastic population that exhibits diverse phenotypes within distinct intracellular environments.^[^ ^8^, ^10^ ^]^ Recent studies have revealed that tumor‐infiltrating neutrophils can manifest both antitumor and protumor phenotypes, exerting influence on tumor immune responses and prognosis through interactions with other immune cells.^[^ ^11^, ^12^, ^13^ ^]^ However, in the field of CC, the exact nature of neutrophils in the TIME remains unclear, let alone cellular communications with other immune cells. Hence, a comprehensive investigation into the roles of neutrophils in CC, particularly throughout the transition from a healthy state to CC, as well as their interplay with other immune cells, is imperative.

In this study, we revealed the three tissue‐specific neutrophils stages during the malignant progression of CC. 1) We discovered that CC patients with elevated neutrophil levels had a higher probability of high‐risk pathological factors. 2) We discovered three tissue‐specific neutrophils stages during the progression of CC, namely, normal cervix‐associated neutrophils (NANs), precancerous‐associated neutrophils (PANs), and tumor‐associated neutrophils (TANs), spanning from tumor defenders (NANs) to instigators (PANs) and ultimately to promoters (TANs), but also emphasized that TANs displayed three malignant biological activities, including angiogenesis, immunosuppression and dysregulated oxidative lipid metabolism to promote malignant progression, highlighting the combination therapy of TANs depletion and anti‐PD1 as a clinical intervention approach to enhance immunotherapy efficacy in CC.

## 2 Results

### 2.1 Clinical Cohort: CC Patients with Elevated Neutrophil Count Had a Higher Incidence of Pathological High‐Risk Factors

In order to investigate the association between neutrophils and clinical characteristics of CC, we conducted a retrospective analysis of 543 CC patients from the Obstetrics & Gynecology Hospital of Fudan University from July, 2024 to July, 2025 (**Table** **1**). Based on the absolute value of normal peripheral blood neutrophils ranging from 1.8 to 6.3 × 1*10^9^, we divided these 543 CC patients into a normal‐neutrophil‐count group and a higher‐neutrophil‐count group (exceeding 6.3 × 1*10^9^). Intriguingly, we found that CC patients with high neutrophil counts had higher incidence of lower‐abdominal pain, parametrial involvement, lymphovascular space invasion, perineural invasion and fallopian tube/ovarian invasion, indicating a close correlation between high neutrophil count and poor clinical presentation in CC patients.

**Table 1:** Analysis of clinical data of cervical cancer patients.

Comparison items	Group (n = 543)		Statistical differences	
Clinical features of cervical cancer	Higher neutrophil count (n = 53)	Normal range neutrophil count (n = 490)	X^2^/t	P value
Age	51.14 ± 10.05	50.04 ± 9.49	0.76	0.833
Low‐abdominal pain	7 (13.21%)	27 (5.51%)	4.828	0.028
Abnormal vaginal discharge	14 (26.42%)	78 (15.92%)	3.745	0.053
Abnormal vaginal bleeding	29 (54.72%)	218 (44.49%)	2.017	0.156
Cervical contact bleeding	20 (37.74%)	207 (42.24%)	0.4	0.527
Vaginal wall involvement	19 (35.85%)	163 (33.27%)	0.143	0.705
Parametrial involvement	13 (24.53%)	45 (9.18%)	11.804	<0.001
Lymphovascular space invasion	39 (73.58%)	284 (57.96%)	4.845	0.028
Deep stromal infiltration	37 (69.82%)	286 (58.37%)	2.599	0.107
Uterine involvement	11 (20.75%)	93 (18.98%)	0.097	0.755
Vaginal margin involvement	5 (9.43%)	35 (7.14%)	0.368	0.544
Perineural invasion	10 (18.87%)	38 (7.76%)	7.329	0.007
Lymph node metastasis	16 (30.19%)	124 (25.31%)	0.596	0.44
Tubal/ovarian invasion	4 (7.55%)	5 (1.02%)	12.499	<0.001

### 2.2 Neutrophils Exhibited High Infiltration Abundance from Normal Cervical Tissue to HSIL and CC in the Single‐Cell Transcriptome

**Figure 1:**
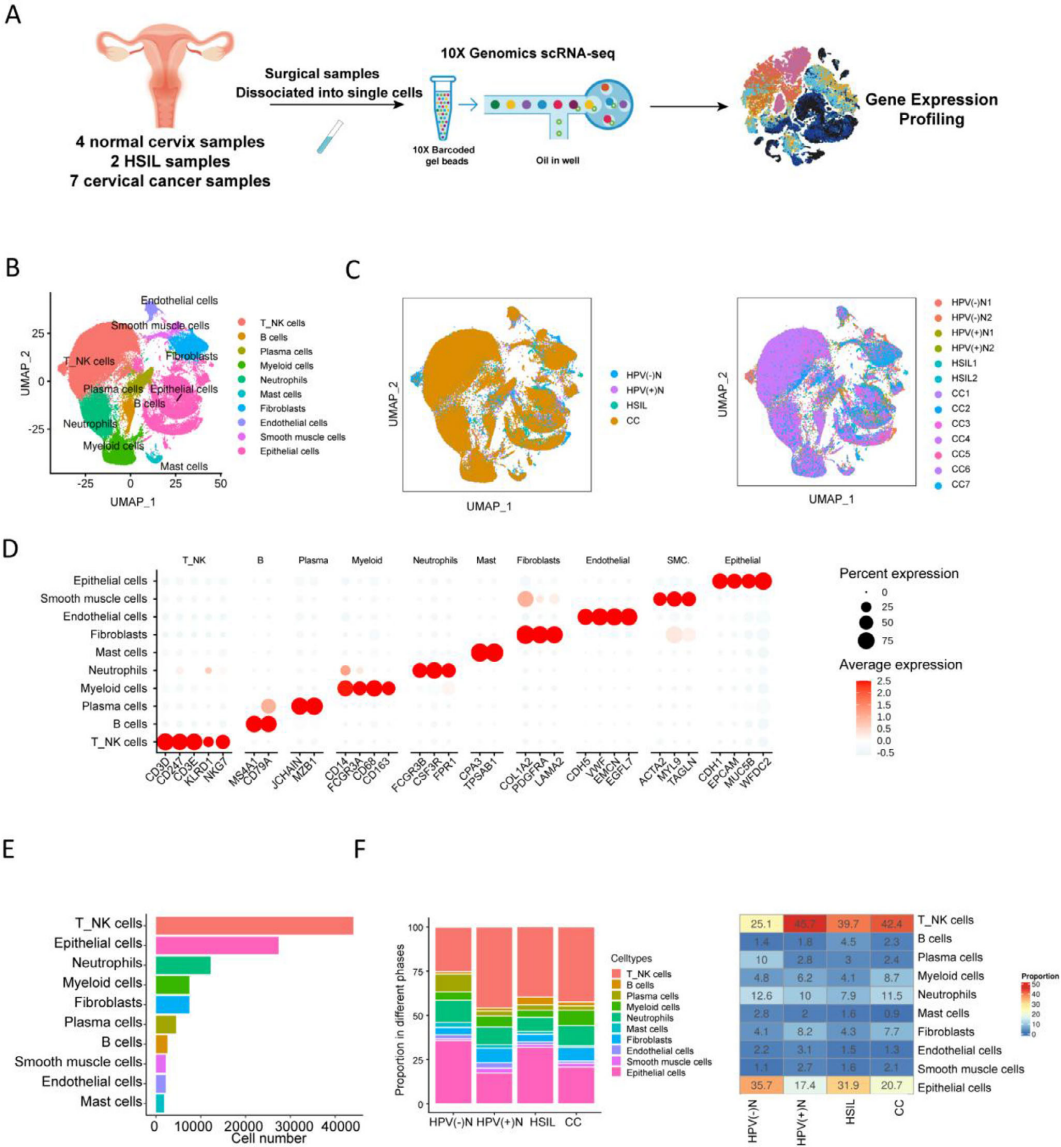
Single‐cell transcriptome landscape of normal cervix tissues, HSIL tissues, and cervical cancer tissues. A) Schematic diagram showing the workflow of the study design and analysis. B) UMAP projection of 111 753 cells from 13 clinical samples consisting of normal cervical tissues, HSIL, and CC tissues that were clustered into 10 cell types. Each dot, which corresponds to a single cell, is colored according to cell type. C) UMAP projection of 111 753 cells in the four disease phases (Left, HPV(−)N, HPV(+)N, HSIL, and CC) and the 13 samples (Right). Each dot, which corresponds to a single cell, is colored according to cell type. D) Average marker gene expression plotted onto the UMAP projection of 10 cell types. The color gradation from grey to red indicates relative expression levels from low to high, respectively. E) Histogram showing the total cell numbers of 10 cell types in 13 samples. F) Histogram (Left) and heatmap (Right) showing the proportions of 10 cell types across different phases in normal cervical tissue, HSIL tissues and CC tissues. HPV(−)N: HPV(−) normal cervix tissue; HPV(+)N: HPV(+) normal cervix tissue; HSIL: high‐grade squamous intraepithelial lesion; CC: cervical cancer.

To reveal alterations in the cervical tissue immune microenvironment during the progression from normal cervical tissue to cancer, we piloted scRNA‐seq on 4 normal cervical tissues, 2 HSIL tissues and 7 CC tissues (**Figure** **1**A). Through standard data processing and quality control procedures, 111753 cells were subsequently reclustered into 10 clusters through principal component analysis (PCA) (Figure 1B,C), annotated by known cell‐type marker genes (Figure 1D): epithelial cells (CDH1, EPCAM, MUC5B, WFDC2), smooth muscle cells (ACTA2, MYL9, TAGLN), endothelial cells (CDH5, EMCN, EGFL7, VWF), fibroblasts (PDGFRA, LAMA2, COL1A1), mast cells (TPSAB1, CPA3), neutrophils (CSF3R, FPR1, FCGR3B), myeloid cells (CD14, FCGR3A, CD68, CD163), plasma cells (JCHAIN, MZB1), B cells (CD79A, and MS4A1) and NKT cells (CD3D, CD247, CD3E, KLRD1, NKG7). The total cell numbers of the 10 cell types are shown in Figure 1E. Among these, the average proportion of neutrophils in 13 samples was 10.9% (Figure 1F; Figure S1A,B, Supporting Information), indicating the infiltration abundance of neutrophils in most samples, which was underestimated previously due to technical limitations and inherent fragility. Thus, in the following analysis, we focused on the role of neutrophils in the progression from normal cervical tissue to HSIL and CC.

### 2.3 Neutrophils Played an Essential Role during the Progression from Normal Cervical Tissue and HSIL to CC

**Figure 2:**
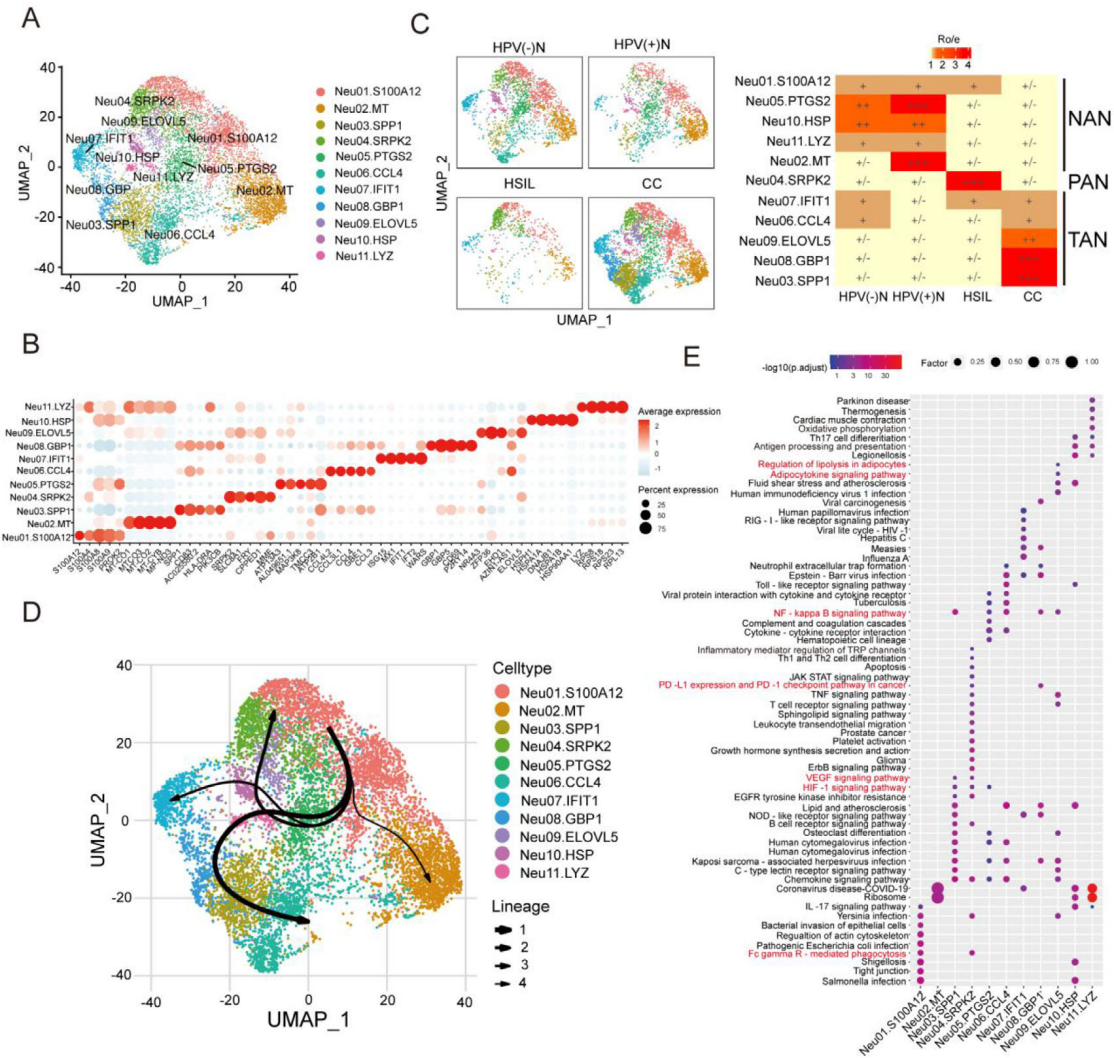
Neutrophil heterogeneity and function in the progression of cervical cancer. A) The UMAP plot showing 11 neutrophil clusters. Each dot, which corresponds to a single cell, is colored according to cell type. B) Average expression of cell type‐specific marker genes in 11 neutrophil clusters. C) UMAP projection of 11 neutrophil clusters in different tissue origins (Left); Tissue preferences of neutrophil clusters in humans, revealed by Ro/e (Right, ratio of observed cell number to expected cell number). D) Trajectory analysis of neutrophil clusters predicted by slingshot. E) Analysis of KEGG functional enrichment of different neutrophil clusters. KEGG: Kyoto Encyclopedia of Genes and Genomes. NAN: adjacent cervix neutrophils; PAN: precancerous‐associated neutrophils; TAN: tumor‐associated neutrophils.

To further investigate the phenotype dynamics of neutrophils in the progression from normal cervical tissue to HSIL and CC, we identified 11 clusters of 8779 neutrophils through unsupervised reclustering (**Figure** **2**A). Based on the specific gene markers of each neutrophil subcluster and the distribution ratio in samples of the three disease stages (normal cervix‐HSIL‐CC), we successfully identified normal cervix associated neutrophils (NANs), precancerous lesion associated neutrophils (PANs) and tumor‐associated neutrophils (TANs). Specifically, five neutrophil clusters were mainly enriched in normal cervical tissues and designated as NANs (S100A12^+^NANs, MT^+^NANs, PTGS2^+^NANs, HSP^+^NANs, LYZ^+^NANs). Another neutrophil cluster was mainly enriched in the HSIL sample and thus designated as PANs (SPRK2^+^PANs). Five neutrophil clusters were mainly enriched in CC samples and thereby designated as TANs (SPP1^+^TANs, CCL4^+^TANs, IFIT1^+^TANs, GBP1^+^TANs, ELOVL5^+^TANs) (Figure 2B,C; Figure S1C, Supporting Information). Meanwhile, trajectory analysis revealed three differentiation pathways from NANs to NANs (lineage 4, mainly concentrated in normal cervical tissues), NANs to PANs (lineage 2, mainly concentrated in HSIL tissues), and NANs to TANs (lineage 1, 3, mainly concentrated in tumor issues), indicating the existence of three tissue‐specific neutrophil stages (Figure 2D). Overall, we identified three tissue‐specific neutrophils stages during CC progression, which encouraged us to further decipher their manifold facets.

### 2.4 NANs and PANs Acted as Tumor Defenders and Tumor Agitators Respectively

#### 2.4.1 CC Defenders: NANs Regulated Immunosurveillance to Prevent Tumorigenesis

**Figure 3:**
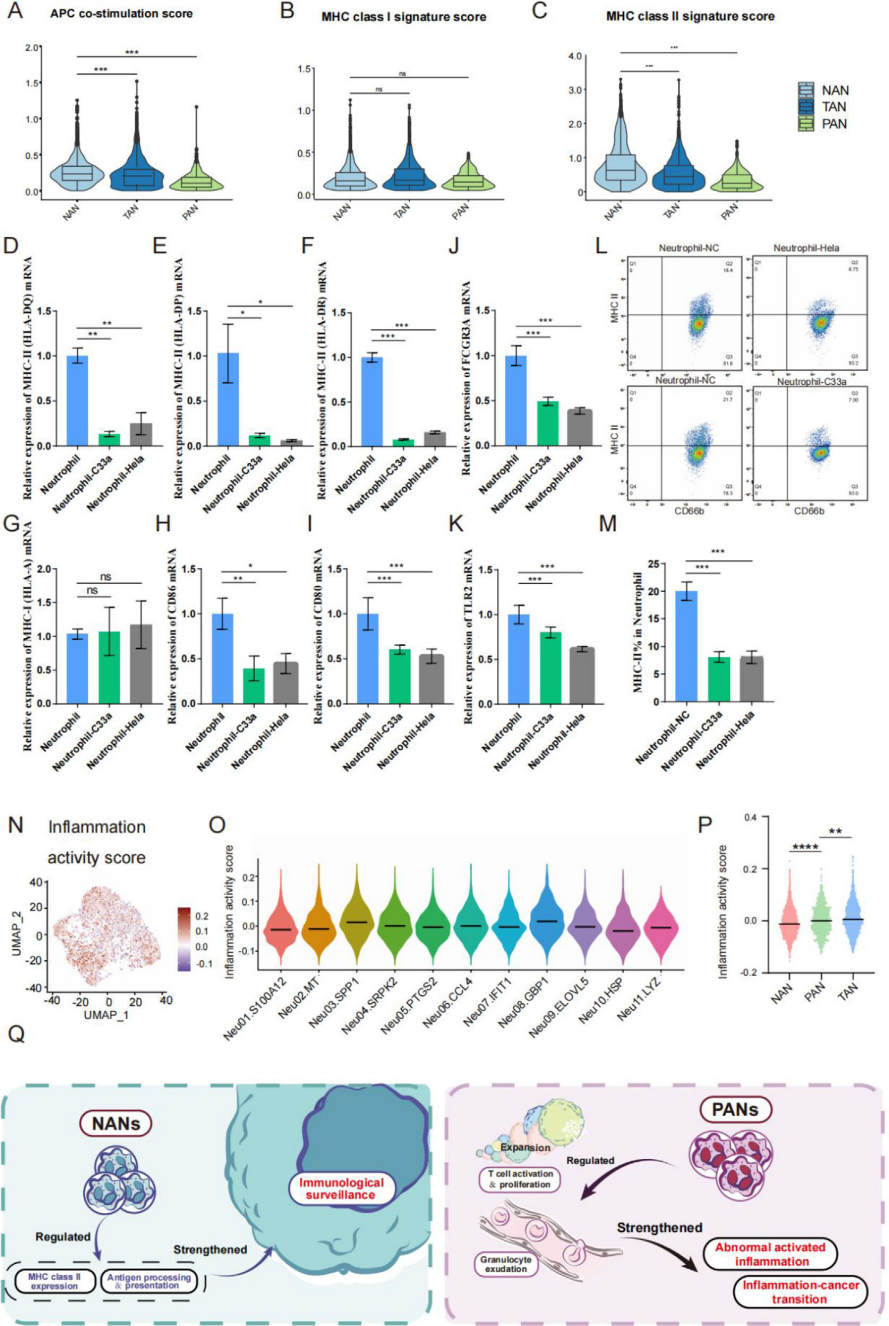
Functional characteristics of NAN and PAN clusters in CC TIME. A) Comparative analysis of APC co‐stimulation scores among NAN, PAN and TAN. B) Quantitative assessment of MHC classI scores among NAN, PAN and TAN. C) Quantitative expression of MHC classII scores among NAN, PAN and TAN. D–I) The expression levels of D) HLA‐DQ, E) HLA‐DP, F) HLA‐DR, G) HLA‐A, H) CD86, I) CD80, J) FCGR3A, and K) TRL2 in neutrophils from healthy peripheral blood co‐cultured with CC cells. In co‐culture system, neutrophils were cultured in upper chambers, and Hela or C33a cell line were cultured in lower chambers in 6 well transwell plates with 0.4 µm pore polyester membrane insert. L) The results of flow cytometry showed the expression level of MHC classII in neutrophils from healthy peripheral blood versus those co‐cultured with Hela or C33a cell line. In co‐culture system, neutrophils were cultured in upper chambers, and Hela or C33a cell line were cultured in lower chambers in 6 well transwell plates with 0.4 µm pore polyester membrane insert. M) The percentage of MHC classII in neutrophils from peripheral blood co‐cultured with CC cells. N) The UMAP projection of inflammation scores of neutrophil cells. O) Comparative abnormal inflammation activation states among neutrophil clusters. P) The abnormal inflammation activation scores of NANs, PANs and TANs. Q) The proposed schematic diagram of NANs and PANs. NANs enhanced the immune surveillance of cervical cancer cells by regulating the expression of MHC classII and antigen presentation. PANs strengthen abnormal activated inflammation and inflammation‐cancer transition by modulating T cell activation and proliferation. Statistical analysis was performed by two‐tailed, unpaired Student's t‐test and one‐way ANOVA, *p < 0.05, **p < 0.01, ***p < 0.001, ns: not significant.

Subsequently, we first focused on the feature and function of NANs among the three tissue‐specific neutrophils stages. According to KEGG analysis, a strong enrichment of FcγR‐mediated phagocytosis, Toll‐like receptor signalling, and antigen processing in NANs was revealed, suggesting an immunosurveillance effect of NANs on tumorigenesis^[^ ^14^, ^15^, ^16^ ^]^ (Figure 2E; Figure S1D, Supporting Information). Besides, we performed APC co‐stimulation score, MHC class I signature score, and MHC class II signature score on NANs, PANs, and TANs, respectively (**Figure** **3**A–C), and found that NANs had the highest APC co‐stimulation score and MHC class II signature score, indicating their antigen processing function. To validate the results of bioinformatic analysis, we co‐cultured neutrophils sorted from healthy human peripheral blood with CC cell lines (Hela, C33a) respectively (Figure S1E, Supporting Information). We noted that NANs which have not been educated by tumors had higher mRNA expression of MHC class II markers (HLA‐DQ, HLA‐DP, and HLA‐DR), FcγR‐mediated phagocytosis marker (FCGR3A), Toll‐like receptor signalling marker (TLR2), antigen processing (CD80, CD86) and higher expression of MHC II molecules through flow cytometry than that educated, indicating that NANs display immune surveillance effects, which may be gradually weakened as tumors progress (Figure 3D–M; Figure S1F, Supporting Information). In general, NANs mainly exerted a pivotal influence on immunosurveillance and maintained immune homeostasis.

#### 2.4.2 CC Agitators: PANs Regulated Abnormal Inflammatory Activation in HSIL

Through KEGG analysis, we discovered that PANs regulated immune activation and inflammatory biological processes, including myeloid leukocyte activation, T‐cell activation and proliferation (Figure 2E; Figure S1D, Supporting Information), suggesting that PANs regulated immune activation in the HSIL stage. Additionally, we conducted an abnormal inflammatory score on the neutrophil subclusters, which reflected the degree of abnormal inflammation in driving inflammation‐cancer transition.^[^ ^17^, ^18^, ^19^ ^]^ The results revealed that the abnormal inflammatory score of PANs was higher than that of NANs and lower than that of TANs (Figure 3N–P), aligning with the chronic inflammatory state caused by persistent HPV infection of the HSIL stage,^[^ ^20^ ^]^ which indicated a trend of dynamic changes in the inflammation‐cancer transition of PANs. In summary, PANs displayed aberrant inflammatory activation, pushing the inflammation‐cancer transition in the HSIL stage (Figure 3Q).

### 2.5 TANs Acted as Tumor Promoters to Potently Drive the Malignancy of CC in Three Ways: Angiogenesis, Immunosuppression, and Disregulated Oxidized Lipid Metabolism

#### 2.5.1 SPP1^+^TANs: Contributing to Angiogenesis in the CC TIME

**Figure 4:**
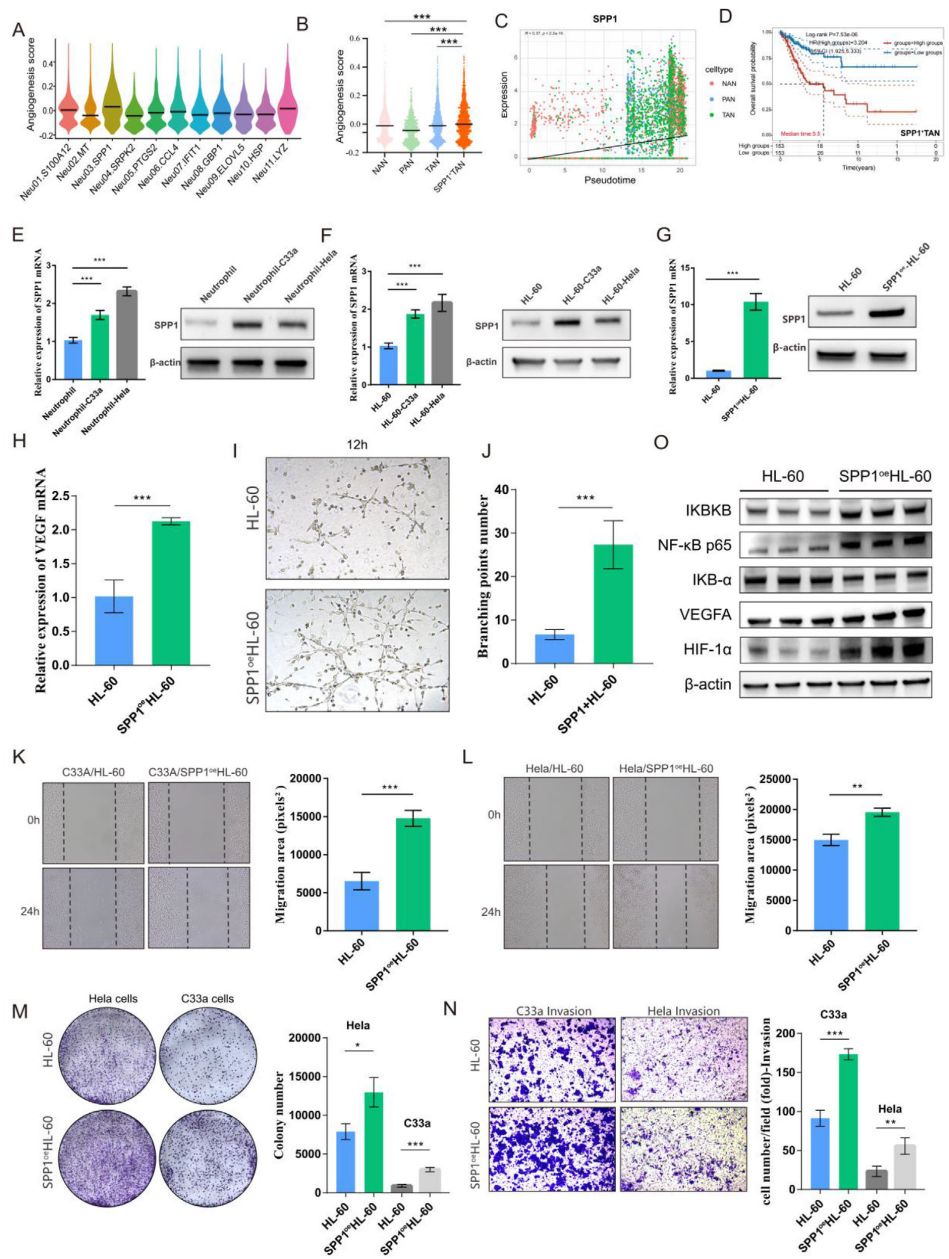
SPP1+TANs contributes to angiogenesis in the CC TIME. A) Angiogenic potential quantification across neutrophil functional subsets. Evolution of SPP1 gene in NAN‐PAN‐TAN pseudotime trajectory B) Comparative angiogenic signature scores among NAN, PAN, TAN and SPP1+TAN subpopulations. C) Evolution of SPP1 gene in NAN‐TAN pseudotime trajectory D) Kaplan–Meier survival curves for the risk model of the SPP1+TANs signature in the TCGA‐CSEC cohort, assessed by LASSO Cox regression analyses. E,F) Comparative analysis of SPP1 expression in peripheral blood‐derived neutrophils and HL‐60 cell line after being co‐cultured with C33a and Hela cells by qRT‐PCR and western blotting (WB). G) Construction of SPP1oe HL‐60 cell line with qPCR and WB validation. H) The expression levels of VEGF in SPP1oe HL‐60/HL‐60 cell line. I,J) The result of tube formation assay of Huvecs after being co‐cultured with SPP1oe HL‐60 and HL‐60 cells at 12 h. K,L) Wound healing assays of C33a and Hela cells migration in lower chamber with SPP1oe HL‐60 / HL‐60 cells co‐culturing in upper chamber. M,N) Colony formation and transwell assay results of C33a and Hela cells after being co‐cultured with SPP1oe HL‐60 cells/HL‐60 cells. O) The WB results of NF‐κB and VEGF/HIF signaling pathway activation in SPP1oe HL‐60 cells. Statistical analysis was performed by two‐tailed, unpaired Student's t‐test and one‐way ANOVA, *p < 0.05, **p < 0.01, ***p < 0.001. CC: cervical cancer; NAN: adjacent cervix neutrophils; PAN: precancerous‐associated neutrophils; TAN: tumor‐associated neutrophils; TCGA: The Cancer Genome Atlas; WB: western blotting.

Given that SPP1 is a hallmark gene of angiogenesis,^[^ ^21^, ^22^ ^]^ and that SPP1^+^TANs regulated the HIF‐1/VEGF signalling pathways (Figure 2E) with the highest angiogenesis score,^[^ ^23^ ^]^ in all neutrophil subclusters (**Figure** **4**A,B), we believe that SPP1+TANs were related to promoting angiogenesis. Notably, the expression level of SPP1 gene gradually increased during the evolution of NAN‐TAN pseudotime trajectory (Figure 4C) and kaplan‒meier analysis of the TCGA‐CESC cohort showed that the SPP1^+^TANs signature was associated with poor overall survival (Figure 4D), therefore we assumed that SPP1^+^TANs might promote angiogenesis in the CC TIME. To validate such hypothesis, 1) first, we co‐cultured neutrophils isolated from peripheral blood of healthy individuals and HL‐60 cells with CC cell lines (Hela and C33a) respectively, and found that the mRNA and protein expression of SPP1 were higher than that of non co‐cultured neutrophils and HL‐60 cells, indicating CC TME induced an increase in SPP1 expression in neutrophils (Figure 4E,F). 2) Second, bioinformatics analysis showed that SPP1^+^TANs exhibited extensive cellular communication with endothelial cell (EC) subclusters, especially tumor‐associated ECs (CXCL12^+^ECs and RGS5^+^ECs) (Figure S2A–F, Supporting Information). Based on this, we conducted multiple immunofluorescence analyses of CC tissues and found co‐localization expression of SPP1+TAN and blood vessels, indicating the pro‐angiogenic function of SPP1+TAN (Figure S2G, Supporting Information). Besides, we constructed SPP1^oe^ HL‐60 cells (Figure 4G) and demonstrated that the VEGF mRNA expression and the ability to promote capillary formation in SPP1^oe^ HL‐60 cells were both obviously elevated compared with the control group, further validating the pro‐angiogenic effect of SPP1 in neutrophils (Figure 4H–J). 3) Furthermore, we found that the migration, proliferation, and invasion abilities of both Hela and C33a were increased after being co‐cultured with SPP1^oe^ HL‐60 cells than HL‐60, suggesting that overexpression of SPP1 in HL‐60 cells can effectively exacerbated the malignant phenotype of CC through promoting angiogenesis (Figure 4K–N; Figure S1G, Supporting Information). 4) Finally, according to KEGG pathway analysis, we observed that SPP1^+^TANs could activate the NF‐κB and HIF/VEGF signaling pathway, thus we further validated that the upregulation of SPP1 indeed activated NF‐κB, and HIF/VEGF signaling pathways using HL‐60 cells (Figure 4O). Overall, SPP1^+^TANs may contribute to angiogenesis through SPP1‐NF‐κB‐HIF/VEGF signaling pathway in the CC TIME and lead to poor overall survival.

#### 2.5.2 GBP1^+^TANs: Facilitating Immunosuppression in the CC TIME

**Figure 5:**
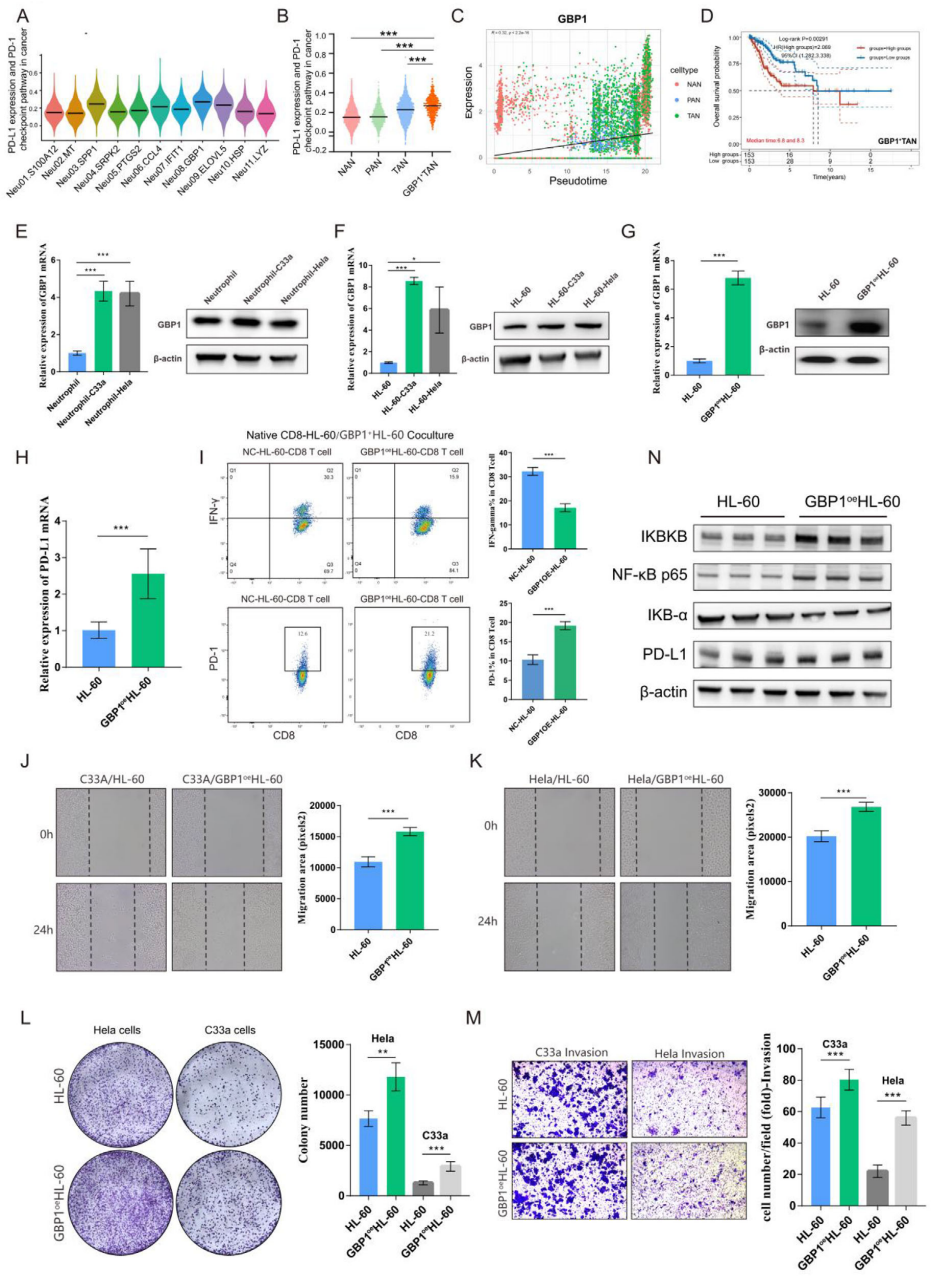
GBP1+TANs contributes to immunosuppression in the CC TIME. A) PD‐L1 expression and PD‐1 checkpoint pathway in cancer score of neutrophil subclusters. B) PD‐L1 expression and PD‐1 checkpoint pathway in cancer score of NANs, PANs, TANs and GBP1+TANs. C) Evolution of GBP1 gene in NAN‐TAN pseudotime trajectory D) Kaplan–Meier survival curves for the risk model of the GBP1+TANs signature in the TCGA‐CSEC cohort, assessed by LASSO Cox regression analyses. E,F) Comparative analysis of GBP1 expression in peripheral blood‐derived neutrophils and HL‐60 cell line after co‐culturing with C33a and Hela cells by qRT‐PCR and western blotting. G) Construction of GBP1oe HL‐60 cell line with qPCR and wb validation. H) The expression levels of PD‐L1 in GBP1oe HL‐60 and HL‐60 cell line. I) The results of PD‐1 and IFN‐ γ expression in naive CD8^+^ T cells after being co‐cultured with HL‐60/GBP1oe HL‐60 cells by flow cytometry. J,K) Wound healing assays of C33a and Hela cells migration in lower chamber with GBP1oe HL‐60/ HL‐60 cells co‐culturing in upper chamber. L,M) Colony formation and transwell assay results of C33a and Hela cells after being co‐cultured with GBP1oe HL‐60/HL‐60 cells. N) The WB results of NF‐κB and PD‐L1 signaling pathway activation by GBP1oe HL‐60 cells. Statistical analysis was performed by two‐tailed, unpaired Student's t‐test and one‐way ANOVA, *p < 0.05, **p < 0.01, ***p < 0.001. WB: western blotting.

Regarding the immune activity of the five TAN subclusters, we discovered that GBP1^+^TANs expressed the highest PD‐L1 expression and PD‐1 checkpoint pathway in cancer score^[^ ^24^ ^]^ among all subclusters (**Figure** **5**A,B) indicating that GBP1^+^TANs might facilitate CC TIME immunosuppression. Moreover, as neutrophils progressed along the NAN‐TAN pseudotemporal axis, GBP1 expression steadily increased (Figure 5C) and kaplan‒meier analysis of the TCGA‐CESC cohort also showed that the GBP1^+^TANs signature was an important risk factor for poor overall survival in CC patients (Figure 5D), suggesting the negative impact of GBP1+TAN in CC TME cannot be ignored. 1) Subsequently, by co‐culturing neutrophils isolated from peripheral blood of healthy individuals and HL‐60 cells with CC cells (Hela and C33a) respectivly, we discovered that the mRNA and protein expression of GBP1 were higher than that of non co‐cultured neutrophils and HL‐60 cells indicating that CC TME induced an increase in GBP1 expression in neutrophils (Figure 5E,F). 2) In addition, bioinformatics analysis revealed extensive crosstalk between GBP1+ TANs and exhausted T cells (PDCD1+CD8^+^ T cells, Tex), mediated by the CCL3‐CCR1 and CCL5‐CCR1 interaction axes, promoting exhausted T cells recruitment, which may engage in immunosuppression in CC TIME (Figure S3A–F, Supporting Information). In view of this, we revealed co‐localization expression of GBP1+TAN and PD‐1+CD8^+^ T cells with multiple immunofluorescence, implying the induction of T cell immune suppression by GBP1+TAN (Figure S3G, Supporting Information). Furthermore, we constructed GBP1^oe^ HL‐60 cells (Figure 5G) and found that the PD‐L1 expression was higher in GBP1^oe^ HL‐60 cells than that in HL‐60 cells, manifesting GBP1 may regulate immune suppression (Figure 5H). Ulteriorly, we sorted naive CD8^+^ T cells from peripheral blood of healthy donors and co‐cultured them with HL‐60/GBP1^oe^HL‐60 cells (Figure S1E, Supporting Information), and observed that the cytotoxicity marker (INF‐γ) decreased and the immunosuppressive marker (PD‐1) increased in CD8^+^ T cells in GBP1^oe^HL‐60 cells‐culture system than that in HL‐60 culture system, further suggesting an immunosuppressive effect of GBP1^+^TANs (Figure 5I; Figure S3H, Supporting Information). 3) Furthermore, we demonstrated that the migration, proliferation, and invasion abilities of both Hela and C33a cells were increased after being co‐cultured with GBP1^oe^ HL‐60 cells than HL‐60 cells, suggesting that overexpression of GBP1 in HL‐60 cells can effectively aggravate the malignant phenotype of CC through promoting immunosuppression (Figure 5J–M; Figure S1G, Supporting Information). 4) Additionally, we revealed GBP1^+^TANs activated NF‐κB signaling pathway based on the KEGG results and further validated that the upregulation of GBP1 activated NF‐κB‐PD‐L1 signaling pathway in HL‐60 cells (Figure 5N). In summary, GBP1^+^TANs may play a prominent role in immunosuppression in the CC TIME through GBP1‐NF‐κB‐PD‐L1 signaling pathway, thereby causing poor overall survival.

#### 2.5.3 ELOVL5^+^TANs: Promoting Abnormally Upregulated Oxidized Lipid Metabolism in CC TIME

**Figure 6:**
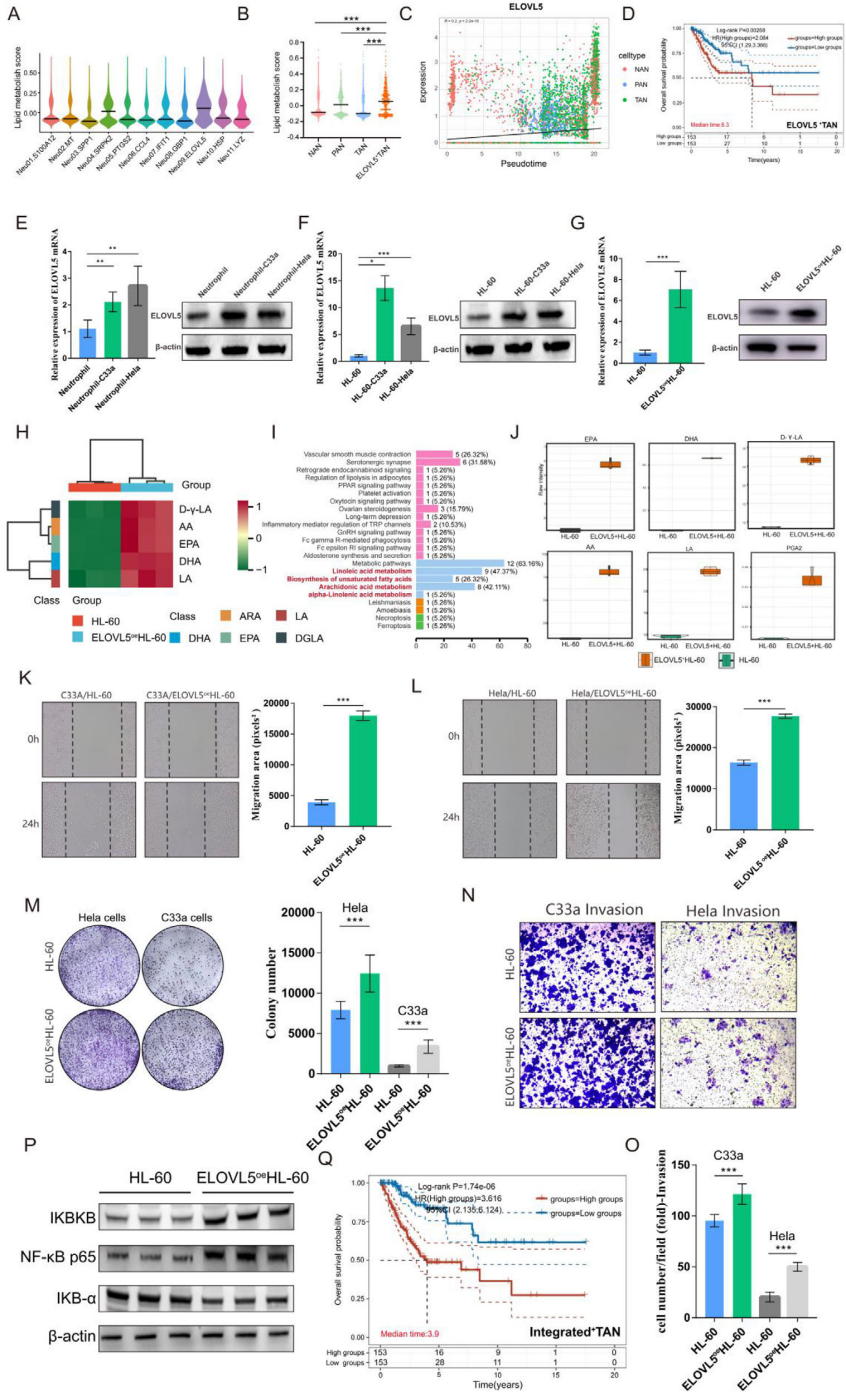
ELOVL5+TANs promotes abnormally upregulated lipid metabolism in the CC TIME. A) Lipid metabolism score of neutrophil subclusters. B) Lipid metabolism score of NANs, PANs, TANs and ELOVL5+TANs. C) Evolution of ELOVL5 gene in NAN‐TAN pseudotime trajectory D) Kaplan–Meier survival curves for the risk model of the ELOVL5+ TANs signature in the TCGA‐CSEC cohort, assessed by LASSO Cox regression analyses. E,F) Comparative analysis of ELVOL5 expression in peripheral blood‐derived neutrophils and HL‐60 cell line after being co‐cultured with hela and c33a cells by qRT‐PCR and western blotting (WB). G) The construction of ELVOL5oe HL‐60 cell line with qPCR and wb validation. H) Heatmap of lipid oxidation metabolites in the ELVOL5oe HL‐60 compared with HL‐60 cells, including DHA, EPA, ARA, DGLA and LA. I) Functional enrichment analysis results of lipid oxidation metabolites in the ELVOL5oe HL‐60 compared with HL‐60 cells. J) Quantitative mass spectrometry results of DHA, EPA, ARA, DGLA and LA in the HL‐60 cells and the ELVOL5oeHL‐60 cells. K,L) Wound healing assays quantifying hela and c33a cells migration in lower chamber with ELVOL5oe HL‐60/ HL‐60 cells co‐culturing in upper chamber. M–O) Colony formation and transwell assay results of hela and c33a cells after being co‐cultured with ELVOL5oe HL‐60 cells/HL‐60 cells. P) The WB results of NF‐κB signaling pathway activation by ELVOL5oe HL‐60 cells Q) Kaplan–Meier survival curves for the risk model of the Integrated+TANs signature in the TCGA‐CSEC cohort, assessed by LASSO Cox regression analyses. Statistical analysis was performed by two‐tailed, unpaired Student's t‐test and one‐way ANOVA, *p < 0.05, **p < 0.01, ***p < 0.001. DGLA: Dihomo‐γ‐Linolenic Acid; AA: Arachidonic Acid; EPA: Eicosapentaenoic Acid; DHA: Docosahexaenoic Acid; LA: Linoleic Acid; WB:western blotting.

Based on KEGG results, we were surprised to identify that ELOVL5^+^TANs regulated adipocytokine signalling pathway and the lipolysis in adipocytes (Figure 2F) and got the highest lipid metabolism score (**Figure** **6**A,B). Besides, along the NAN‐TAN developmental trajectory, ELOVL5 levels were incrementally upregulated (Figure 6C). Given that the upregulation of lipid metabolism could contribute to rapid tumor growth,^[^ ^25^ ^]^ kaplan‒meier analysis of the TCGA‐CESC cohort indicated that the ELOVL5^+^TANs signature led to poor overall survival in CC (Figure 6D), we hypothesised that ELOVL5^+^TANs may accelerate CC progression by dysregulating lipid metabolism. 1) After co‐culturing neutrophils and HL‐60 cells with CC cells (Hela and C33a), respectively, we found that the mRNA and protein expression of ELOVL5 were higher than that of non co‐cultured, indicating CC TME induced an increase in ELOVL5 expression in neutrophils (Figure 6E,F). 2) In addition, we constructed ELOVL5^oe^ HL‐60 cells (Figure 6G), and quantitative mass spectrometry data of oxidized lipids also showed that ELOVL5^oe^ HL‐60 cells produced more long‐chain unsaturated fatty acid metabolites (LC‐PUFAs): ([dihydroxymethyl ‐ γ ‐ linolenic acid] D ‐ γ ‐ LA, [arachidonic acid] AA, [eicosapentaenoic acid] EPA, [docosahexaenoic acid] DHA, [linoleic acid] LA) than HL‐60 cells, which can serve as energy sources for TAN to maintain longer activity and functional performance. Meanwhile, enrichment analysis showed the most significant correlation with lipid metabolism pathways, further indicating that ELOVL5 may upregulate oxidative lipid metabolism (Figure 6H–J). 3) Furthermore, we demonstrated the pro‐tumor phenotype of ELOVL5^oe^ HL‐60 cells that the migration, proliferation, and invasion abilities of both Hela and C33a cells were increased after being co‐cultured with ELOVL5^oe^ HL‐60 cells(Figure 6K–O), suggesting that overexpression of ELOVL5 in HL‐60 cells can effectively accelerate the malignant phenotype of CC through promoting upregulated oxidized lipid metabolism. 4) Moreover, as ELOVL5^+^TANs activated NF‐κB signaling pathway based on the KEGG results, we further validated that the upregulation of ELOVL5 activated NF‐κB signaling pathway through western blot (Figure 6P). To summarize, ELOVL5^+^TANs might contribute to aberrantly upregulated lipid metabolism, thereby promoting tumor progression and resulting in a poor CC prognosis.

**Figure 7:**
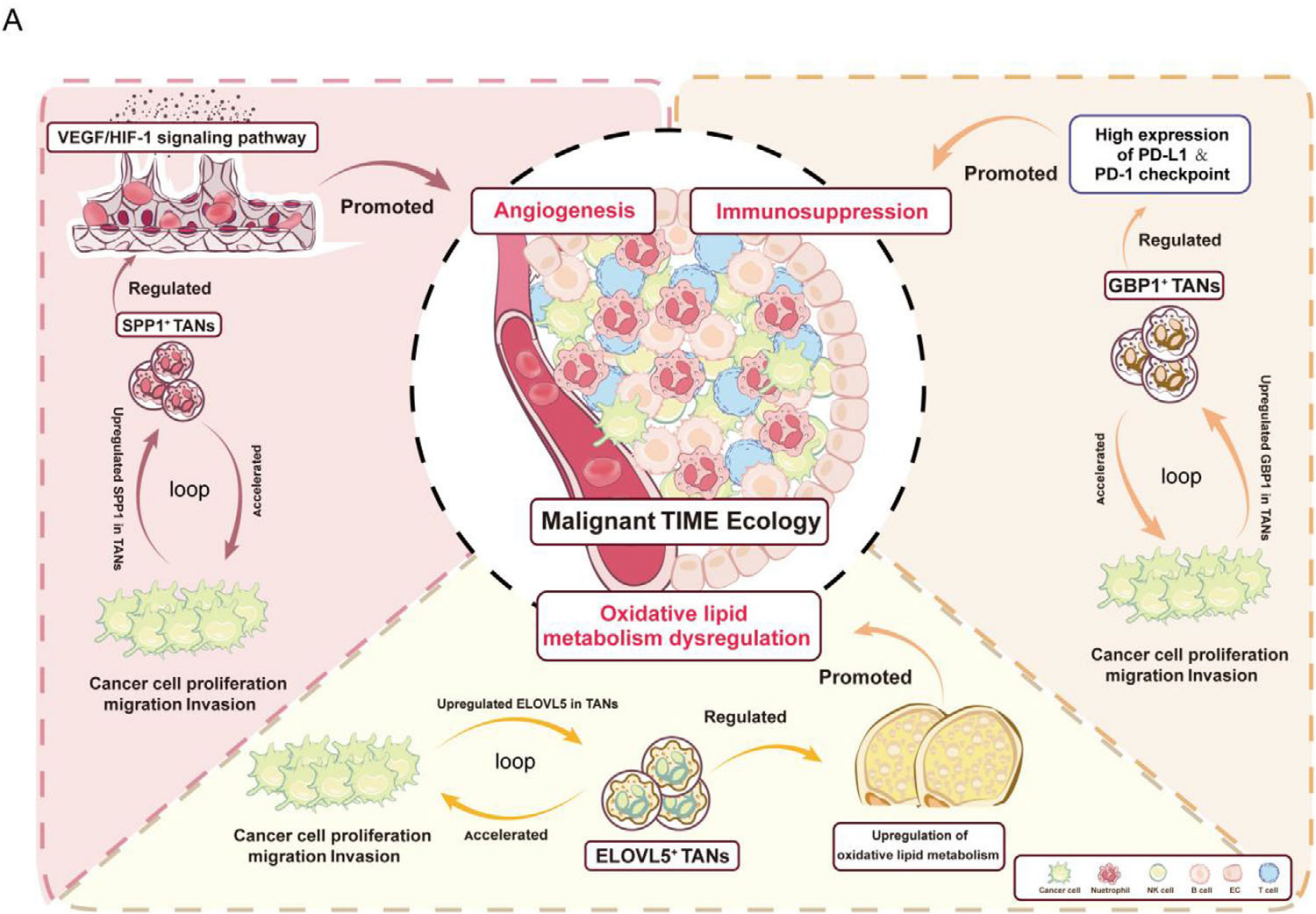
A) The functional schematic diagram of TANs. TANs impact the TIME of CC through three aspects: angiogenesis (SPP1/NF‐κB/VEGF axis), immunosuppression (GBP1/NF‐κB/PD‐L1 axis) and metabolism (ELOVL5/NF‐κB axis).

In summary, we comprehensively analysed the polytropic effects of TANs in the TIME of CC from the three perspectives of angiogenesis (SPP1‐NF‐κB‐HIF/VEGF axis), immunosuppression (GBP1‐NF‐κB‐PD‐L1 axis), and aberrant lipid metabolism (ELOVL5‐NF‐κB axis) (**Figure** **7**A). The signatures of the three TANs contribute to poor prognosis. And further, we found that the integrated signature of three TANs is more valuable for predicting prognosis than each one individually (Figure 6Q), suggesting that the combination therapy targeting all the three TAN subclusters holds promising therapeutic potential.

### 2.6 Preclinical Exploration of Combining Anti‐TANs Therapy (anti‐Ly6g) and Anti‐PD1 Therapy In Vivo Xenografts as a Promising Means to Improve Immunotherapy Efficacy of CC

**Figure 8:**
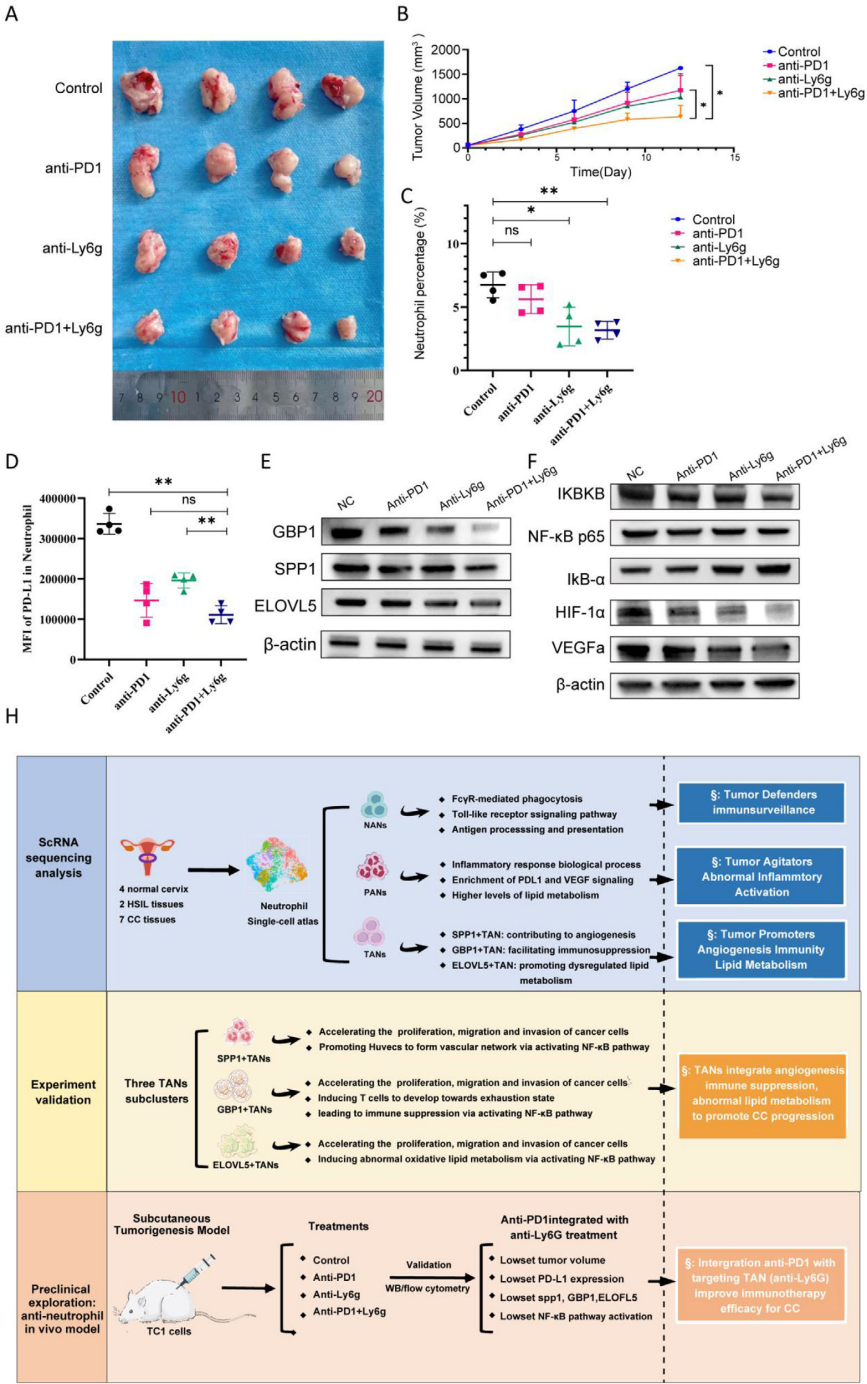
Neutrophil deletion further enhanced the treatment efficacy of immunotherapy in CC animal models. A) Photographs of TC1 tumors by the indicated treatment. The tumors were removed from C57BL/6 mice on day 12 after TC1 cell injection (n‐4 for each group in Figure 7A: isotype as control group (Control), anti‐PD1, anti‐Ly6g, and anti‐PD1+anti‐Ly6g. B) Average tumor growth curves of TC1 tumors in mice that underwent different treatments. Error bar: mean value + SD (n=4 for each treatment group). C,D) Scatter plots showing the neutrophil percentage and the expression of PD‐L1 in neutrophils from CC tissues of C57BL/6 mice bearing TC1 tumors after different treatment regimens through flow cytometry. Error bar: mean value with 95% CI (n‐4 for each treatment group). E,F) Sorting neutrophils from tumor tissues of mice treated with four different methods, and detect the expression of SPP1, GBP1, ELOVL5, and NF‐κB signaling pathway by WB analysis. G) Flow chart and critical conclusions of the current study. Statistical analysis was performed by two‐tailed, unpaired Student's t‐test and one‐way ANOVA, *p < 0.05, **p < 0.01, ***p < 0.001, ns; not significant; WB: western blotting.

Based on the key protumorigenic role of TANs, we speculated about their potential as therapeutic targets. Consequently, we constructed a subcutaneous tumorigenesis model in C57BL/6 mice with murine CC cells (TC1) to evaluate the effectiveness of anti‐Ly6g (suppressing TANs in mice) treatment (**Figure** **8**A). Considering the robust immunosuppressive effects of TANs, we also attempted to combine anti‐Ly6g with anti‐PD1 therapy. Remarkably, the combined therapy group had the best effect on tumor shrinkage, suggesting that anti‐ly6g combined with anti‐PD1 therapy can effectively improve the efficacy of anti‐PD1 alone for CC (Figure 8B,C). Furthermore, the expression of SPP1, GBP1, ELOVL5, PD‐L1 in neutrophils exhibited a most substantial decrease after the combination therapy (Figure 8D,E), verifying that the combination therapy can effectively suppress TANs. Besides, the combination therapy also had the best inhibitory effect on NF‐κB pathway, VEGF/HIF‐α signaling pathway, and PD‐L1 expression, thereby exerting an antitumor effect (Figure 8F). Overall, these findings highlighted that the combination therapy showed a more favorable immunotherapy efficacy regarding antitumor effects than anti‐PD1 monotherapy, providing novel clinical insights.

## 3 Discussion

Addressing the imperative challenge of enhancing the responsiveness of immunotherapy in the context of CC necessitates deciphering the intricate and highly heterogeneous TIME, wherein the roles of neutrophils as potent modulators of tumor progression has garnered increasing recognition, owing to their remarkable phenotypic plasticity.^[^ ^26^ ^]^ However, unravelling the true essence of neutrophils within the TIME of CC remains an enigmatic endeavor. In this study, we revealed that CC patients with elevated neutrophil count have a higher incidence of high‐risk pathological factors, suggesting a correlation with poorer prognosis. Furthermore, we elaborated on the three tissue‐specific neutrophils stages (NANs, PANs, TANs) throughout the progression from normal cervical tissue to HSIL and eventually to CC. In the normal state, NANs acted as defenders to primarily engage in immune surveillance. In the HSIL stage, PANs acted as agitators to activate aberrant inflammation, thus promoting the inflammation‐cancer transition. In CC progression, a malignant positive feedback loop emerged between tumor cells and TANs. Tumor cells reprogrammed TANs to overexpress SPP1, GBP1, and ELOVL5. TANs subsequently played a comprehensive role in CC progression by promoting angiogenesis (SPP1‐NF‐κB‐HIF/VEGF), immunesuppression (GBP1‐NF‐κB‐PD‐L1), and dysregulating oxidative lipid metabolism (ELOVL5‐NF‐κB). These TAN‐derived pathological effects amplified tumor malignancy, which reciprocally stimulates tumor cells to further educated TANs to reprogram, creating a self‐amplifying “tumor cell‐TANs” cascade, ultimately accelerating CC progression and poor prognosis. Notably, in vivo experiments substantiated that the combination of anti‐PD1 and anti‐Ly6g therapy yielded superior immunotherapy efficacy than anti‐PD1 monotherapy for CC targeting TANs (Figure 7G). In addition, combination therapy also has the best inhibitory effect on the SPP1, GBP1, ELOVL5, and NF‐κB signaling pathway activation in TANs. Overall, our study presented a comprehensive landscape of neutrophils throughout the progression of CC, providing critical clues for targeting TANs to improve immunotherapy efficacy for CC.

Currently, endeavors to enhance the efficacy of immunotherapy predominantly centre around T‐cells and immune checkpoints, with limited understanding of the capacity of myeloid cells, exemplified by neutrophils, to modulate immunotherapy efficacy within the TIME.^[^ ^27^, ^28^ ^]^ Meanwhile, the phenotypic states of neutrophils remain ambiguous throughout the entirety of CC progression. Strikingly, in this study, during the normal stage, we demonstrated that NANs exploited antigen processing ability by MHC class II, and this ability will decrease with the education of tumors. Therefore, understanding how to effectively sustain the characteristics of NANs may have instructive implications for the prevention of CC. Regarding the HSIL stage, we observed that the high levels of abnormally activated inflammation in PANs fell within an intermediate range, situated between NAN and TAN, suggesting its potential role in further promoting the inflammation‐cancer transition. Hence, developing specific interventions targeting PANs may delay or even reverse the progression of CC.

Regarding the CC stage, we identified that SPP1+TANs contributed to angiogenesis via SPP1‐NF‐κB‐VEGF axis, GBP1+TANs contributed to immunosuppression via GBP1‐NF‐κB‐PD‐L1 axis, whereas ELOVL5+TANs contributed to lipid metabolism dysregulation by ELOVL5‐NF‐κB axis. A recent study also found that a subpopulation of neutrophils highly expressing CD71 is the main executor of immune suppression in brain tumor models. These types of cells preferentially aggregated in the hypoxia area of tumors, exhibiting vigorous glucose metabolism and lactate accumulation, ultimately inducing immune suppression.^[^ ^29^ ^]^ Interestingly, another study on triple‐negative breast cancer found that high expression of ELOVL5 in tumor cells promotes the synthesis of arachidonic acid. After neutrophils uptake arachidonic acid, they are “reprogrammed” into an immunosuppressive phenotype, forming a vicious “tumor cells‐neutrophils” cycle.^[^ ^30^ ^]^ In our study, we observed that increased ELOVL5 and immunosuppression in TANs can conversely act on tumor cells, promoting a “TAN‐tumor cell” vicious cycle, thereby further refining this interactive loop. These TANs‐mediated pro‐tumorigenic effects not only accelerate tumor growth and immune evasion but also reinforce the proliferation of tumor cells, thereby perpetuating the self‐amplifying cycle. This vicious intercellular crosstalk ultimately correlates with accelerated disease progression and worsened clinical outcomes in CC patients. There is still a limitation in this study regarding the other two TAN subgroups, the CCL4+ TANs subcluster, implicated in cytokine secretion and immune cell recruitment via chemotaxis, was not extensively explored here as its functional phenotype is already well‐characterized in the literature.^[^ ^11^ ^]^ Furthermore, while IFIT1+TANs were strongly associated with viral responses, its potential link to specific HPV oncogenic viruses within the TME remained uninvestigated in this work. Elucidating these aspects represents an important direction for future research. Based on these findings, we proposed a CC ecosystem affected by TANs, which broadens our understanding of the TIME and provides new insights for potential targeted therapeutic strategies for TANs.

Targeting TANs for antitumor therapy has shown promising results in clinical trials and basic research across various cancers;^[^ ^11^, ^31^, ^32^, ^33^ ^]^ yet, in the field of CC, it is still ambiguous. In this study, we conducted a preclinical exploration of TANs‐targeted therapy for CC and confirmed that inhibiting TANs can effectively suppress tumor progression and that the combination of anti‐Ly6g and anti‐PD‐1 therapy can result in superior immunotherapy efficacy compared to either monotherapy. In addition, combination therapy also has the best inhibitory effect on the SPP1, GBP1, ELOVL5, and NF‐κB signaling pathway activation in TAN. Considering that clinical trials evaluating the effectiveness of TANs inhibitors in combination with immune checkpoint blockade (anti‐PD1) for CC treatment have not yet been undertaken, we propose the formulation and execution of clinical trials targeting CC patients guided by our preclinical findings. In summary, we found that combining anti‐PD‐1 immunotherapy with TANs‐targeted therapy could be a promising strategy to improve the clinical challenges of poor immunotherapy efficacy.

Generally, we revealed that CC patients with elevated neutrophil count have a higher incidence of high‐risk pathological factors, suggesting a correlation with poorer prognosis. Besides, we illuminated the three tissue‐specific neutrophils stages (NANs, PANs, TANs) across the continuum from normal cervical tissue to HSIL and finally to CC. In the normal state, NANs act as defenders to inhibit tumor initiation. During the HSIL stage, PANs act as agitators to trigger the inflammation‐cancer transition. In the CC stage, a self‐reinforcing tumor cell‐TANs loop drives cervical cancer progression. Tumor cells reprogrammed TANs to upregulate SPP1, GBP1, and ELOVL5, which activated NF‐κB signaling to fuel angiogenesis (SPP1/HIF/VEGF), immunosuppression (GBP1/PD‐L1), and lipid metabolic dysfunction (ELOVL5). These TAN‐mediated effects reciprocally enhanced tumor aggressiveness, further amplifying TAN reprogramming in a mutually reinforcing cycle that accelerated CC progression and poor outcomes. Remarkably, the results of our in vivo experiments validated that the anti‐Ly6g, anti‐PD1, and combination of anti‐PD1 and anti‐Ly6g treatments all attenuated tumor progression. Notably, the combination of anti‐PD1+anti‐Ly6g yielded superior immunotherapy efficacy compared to anti‐PD1 monotherapy for CC. In addition, combination therapy also has the best inhibitory effect on the SPP1, GBP1, ELOVL5, and NF‐κB signaling pathway activation in TAN. In conclusion, we comprehensively elucidated the existence of three tissue‐specific neutrophils stages in the progression from normal cervical tissue to HSIL and finally to CC, encompassing roles as defenders, instigators and promoters, highlighting the potential significance of targeting TANs as a viable clinical intervention strategy to enhance the efficacy of immunotherapy in the treatment of CC.

## 4 Experimental Section

### Human Specimens

A total of 5 formalin‐fixed, paraffin‐embedded CC tissues for multicolor immunohistochemistry (IHC) staining were collected from the tissue bank of the Obstetrics and Gynaecology Hospital of Fudan University, Shanghai, China, under the approval of Ethics Committee (2020‐22; 2025‐128) with informed consent. CC samples were obtained during primary treatment of surgical resection. More detailed clinicopathological information of these 5 samples were listed in Table S1 (Supporting Information).

### scRNA‐Seq Sample Collection

To acquire transcriptomic profiles during the malignant transition of the cervix, we obtained 10x Genomics scRNA‐seq data from 13 cervical tissue samples that were previously generated by our group (Gene Expression Omnibus accession number: GSE197461^[^ ^34^ ^]^ and GSE208653^[^ ^35^ ^]^) including 4 normal cervical tissue samples, 2 HSIL samples obtained by colposcopy biopsy and 7 CC tissues (5 squamous cell carcinoma [SCC] samples and 2 adenocarcinoma [ADC] samples) by surgical resection with pathological diagnosis. All 7 patients with CC were primarily treated preoperatively at FIGO stages I‐II. The exclusion criteria were as follows: (1) serious concomitant systemic disorder, (2) history of chemotherapy or radiotherapy, (3) history of malignant tumors other than CC, and (4) evidence of distant metastasis. The detailed clinical characteristics of the 13 samples are presented in Table S2 (Supporting Information).

### Data Pre‐Processing and Quality Control

For unique molecular identifier (UMI) counting of sequencing data, feature‐barcode matrices were obtained by aligning reads to the human genome (GRCh38 Ensemble: version 100) using CellRanger v5.0.1. Further analysis was conducted using the Seurat package (version 4.1.2 https://satijalab.org/seurat/). In a quality control step, cells were considered as ambient droplets or stressed cells with low quality, if the number of expressed genes was < 200 or if the mitochondrial UMI rate was >10%, and these cells were removed for further analyses.

### Cell‐Type Identification and Sub‐Clustering Analysis

For cell clustering and annotation of major cell types, read counts were normalized across all cells from the 13 samples, followed by scaling manipulation and converting to log scale with the Seurat function “NormalizeData”. Then, the normalized values were used to select highly variable genes (HVGs) with the “FindVariableFeatures” function. Furthermore, the expression profiles of HVGs were converted to z‐scores using the function “ScaleData”. Principle components (PCs) were estimated based on the selected HVGs with the function “RunPCA”, and the first 15 PCs were selected for downstream analysis. The two functions of “FindNeighbors” and “FindClusters” were implemented to find clusters of expression‐similar cells with empirically set resolutions. Differentially expressed genes (DEGs) for each cluster relative to the remaining clusters were identified by the function “FindAllMarkers”. To annotate cell identity of the clusters, we curated a list of canonical gene markers for various cell types, including 13 major types. A cluster was assigned as a canonical identity based on enriched expression of the marker genes, which also listed in the DEGs identified. For sub‐clustering analysis and subtype annotation, extracted cell subset for a target major cell type was performed as was done in the global map above with reduction and cluster parameters of finer resolution to capture more local structure. Marker genes were calculated analogously to the global cluster analysis with default settings, and for each subtype the top fold‐change enriched gene marker with classical function annotation were used to label the subtypes.

### Identification of Differentially Expressed Genes (DEGs) and Function Enrichments

For identification of inter‐group DEGs, Seurat function “FindMarkers” was applied to calculate between the compared groups in t‐test model. The genes were further filtered for gene ontology biological processes (GOBP) analysis at a minimum fold change of 0.25 (avg_logFC) and a maximal p value of 0.05. The “enrichGO” function within the clusterProfiler (v3.14.3) was used for GOBP and KEGG function enrichment. Function enriched with Benjamini–Hochberg corrected two‐sided P‐value < 0.05 were considered as robust results for further visualization.

### Module Score Analysis of Function Processes

Enrichment scores of each cell in the targeted cell population were estimated using the Seurat function “AddModuleScore”, feed with a list of collected genes from literatures or reference GO terms of interest. Briefly, each score was generated by calculating total gene expression for each of the analyzed genes and separating them into 25 bins of similar expression. For every gene in each target signature, 100 “control” genes were selected from its corresponding bin and added to a “control” gene set. The resulting “control” gene sets contained and equivalent expression distribution as the target signature and its average represents an equivalent sampling of 100 genes of equal size to the target gene set. The expression of genes in the target gene set and the “control” gene set were averaged across each cell to generate a target score and control score. The cell's score for the target gene set is the difference between the target score and control score.

### Cell–Cell Interactions

Cell‐cell interactions (CCI) between assigned cell population of different comparisons were inferred using CellChat (version 1.1.3). The CellChat database (CellChatDB) is retrieved at http://www.cellchat.org/cellchatdb/. CCI analyses were performed using the log‐transformed normalized gene counts by default. Different group data used for inter‐group comparisons was feed to CCI analysis workflow, and a merged data object was created by function merge CellChat for further identification of significant CCI changes between the compared groups. The function compareInteractions and netVisual_diffinteraction were used to compare aggregated communication probabilities for compared groups.

### Developmental Trajectory

Slingshot (version 2.8.0) were used to infer cell‐state trajectories among the cell subtypes of interest. Slingshot was run on the UMAP embedding space from previous sub‐clustering analysis. The resulting trajectories and pseudotimes among cell subtypes were retrieved and processed for visualization by ggplot2 (version 3.3.3).

### Tissue and Cancer Type Enrichment of Clusters

To estimate the tissue preference of different cell types, the ratio of observed to expected cell numbers in each cluster (Ro/e) was calculated by the chi‐square test. We first generated a contingency table of the cell clusters of different groups, then applied chi‐squared test to evaluate whether the distribution of the cell clusters across conditions significantly deviates from random expectations.

### TCGA Public Dataset Analysis

RNA‐seq data, clinicopathological information, and survival data of the CESC dataset from the TCGA database were downloaded from Xena (https://xena.ucsc.edu/). The risk models were established using the LASSO Cox regression analysis of SPP1^+^TANs signature, GBP1^+^TANs signature, ELOVL5^+^TANs and Integrated^+^TANs signature, signature based on TCGA‐CESC cohort. Kaplan–Meier analyses and log‐rank test were conducted using the powerful online tool Assistant for clinical bioinformatics (https://www.aclbi.com/) to compare survival differences between patients in the high‐risk and low‐risk subtypes. The time‐dependent receiver operating characteristic (ROC) curves and corresponding areas under the curve (AUC) values were utilized to assess the prognostic value of SPP1^+^TANs signature, GBP1^+^TANs signature, ELOVL5^+^TANs and Integrated^+^TANs signature risk model based on TCGA‐CESC cohort.

### Multicolor IHC Staining

To elucidate the spatial localization of TANs, CD8^+^ T cells and the cellular communication, we conducted multicolor IHC staining assays using the Multiplex IHC kit (mIHC‐3271‐6, Absin, Shanghai, China) according to the manufacturer's instructions. The slides were incubated with a blocking antibody diluent at room temperature (25 °C) for 10 min and subsequently incubated overnight at 4 °C with primary antibodies applied sequentially. The slides were subsequently incubated with a secondary antibody (HRP polymer, anti‐mouse/rabbit IgG; Abcam, Cambridge, UK) at room temperature for 10 min. Next, a fluorophore (tyramide signal amplification plus working solution) was applied to the sections, followed by microwave heat treatment. The nuclei were stained with DAPI (Beyotime, Shanghai, China) after all antigens were labeled. Multispectral images of each stained slide were captured using the Mantra system (PerkinElmer, Waltham, MA, USA). The primary antibodies used listed in Table S3 (Supporting Information).

### Animal Experiments

All animal experiments were approved by the Fudan University Animal Care and Use Committee. Female C57BL/6 mice (5‐6 weeks old) were purchased from the Laboratory Animal Center of the Shanghai Institutes for Biological Sciences (Shanghai, China) and housed in a pathogen‐free environment. Ethical batch number: 2024‐FCKYY‐082 (Ethics Committee of the Obstetrics and Gynaecology Hospital, Fudan University). To establish ectopic tumors, 1 × 10^5^ TC‐1 cells (in 200 µL of PBS) were subcutaneously injected into the right shoulders of the C57BL/6 mice. When the tumor volume reached ≈50 mm^3^ (denoted as day 0), the tumor‐bearing mice were randomly divided into 4 groups (n = 4 in each group): control, anti‐PD‐1, anti‐Ly6g and anti‐PD1+Ly6g groups. The transplant recipients received 200 µg of anti‐PD‐1 (Cat.No.BE0146, Bioxcell, West Lebanon, N.H, USA) and, twice a week × 4 doses (Day 3, Day 6, Day 9, and Day 12) through intraperitoneal injection. The anti‐Ly6G antibody (1A8, Bio X Cell) or IgG2a Isotype control (2A3, Bio X Cell) at a dose of 12.5 µg per 100 µl PBS was administered daily through intraperitoneal injection from day 0 to 12. Tumor size was monitored every 3 days and measured at the time of sacrifice, and the volume was calculated as (1/2 × length × width^2^). Tumor tissues was collected from each mouse for flow cytometry analysis.

### Flow Cytometry

Single‐cell mice tumor tissue suspensions, neutrophils and native CD8^+^ T cells were washed twice with PBS and incubated for 30 min in the dark with antibodies against specific surface proteins. To detect intracellular proteins, cells were stimulated for 6 h with 10 µg/mL brefeldin A and 2 µmol/L ionomycin (Absin, Shanghai, China) before staining. The cells were then fixed and permeabilized using a fixation/permeabilization wash buffer (BioLegend, San Diego, CA, USA) and stained with antibodies for 30 min in the dark. All antibodies used for flow cytometry are listed in Table S4 (Supporting Information). All samples were run on a CytoFLEX platform (Beckman Coulter, Bria, CA, USA) and analyzed using the FlowJo version 10.8 software.

### Cell Culture

HL‐60 (RRID:CVCL_0002), Hela (RRID:CVCL_0030), C33a (RRID:CVCL_1094) and human umbilical vein endothelial cells (HUVECs, RRID:CVCL_9Q53) cell lines were obtained from the Cell Bank of Shanghai Institute of Biotechnology, Chinese Academy of Sciences (Shanghai, China). Neutrophils were autonomously harvested from the blood of healthy donor through a process involving CD66b Biotin antibody (Biolegend, 305120) staining, the addition of biotin magnetic beads, and sorting using MS columns (Miltenyi Biotec, 130‐042‐201). Native CD8^+^ T cells were autonomously harvested from the blood of healthy donor through EasySep ™ Human Naive CD8^+^ T cells cell sorting antibody kit (stemcell, 17968). HL‐60, C33a, neutrophils and native CD8^+^ T cells were treated with 10–20% FBS (Gibco) and antibiotics (Beyote, Penicillin, 100U/ml); Streptomycin (0.1mg/ml) RPMI‐1640 medium (Gibco). Native CD8^+^ T cells were additionally activated and cultured with IL‐2, CD3/CD28 Dynabeads (STEMCEL Technologies).

Hela cells were cultured in DMEM medium with 10% FBS and antibiotics. HUVECs were propagated in endothelial cell medium (ECM; ScienCell, USA) supplemented with 10% FBS (ScienCell), 1% endothelial cell growth supplement (ScienCell), and 1% penicillin and streptomycin (ScienCell). In co‐culture experiments, we used 24 well and 6 well transwell plates with 0.4 µm pore polyester membrane insert (Corning), in which HL‐60 cells, neutrophils huvecs and naive CD8^+^ T cells were cultured in upper chambers, and CC cell lines (hela and c33a cells) were cultured in lower chambers.

### Overexpression of Genes in HL‐60 Cells

The ORFs of human genes SPP1, ELOVL5 and GBP1 were seamlessly cloned into pLV3‐CMV‐3 × FLAG‐CopGFP Puro using cloning technology; Overexpression plasmid, co‐transfection of psPAX2 and pMD2. G into 293T cells for packaging lentivirus; 48 h after viral infection with HL‐60, stable overexpression cell lines were obtained by screening with 1ug/mL puromycin.

### Real‐Time RT‐PCR (qPCR) Analysis

Total RNA was extracted with TRIzol (Invitrogen). 1 mg RNA was used as template for cDNA conversion with HiScript III RT SuperMix (+gDNA wiper) (Vazyme). Real‐time RT‐PCR analysis of mRNA expression was performed in triplicate with AceQ Universal SYBR qPCR Master Mix (Vazyme). Gene expression was normalized to β‐actin as endogenous control.

### Western Blotting

Total protein was extracted using RIPA buffer (epizyme, China) with protease inhibitor cocktail (Roche Applied Science, Switzerland), and quantitated by bicinchoninic acid (BCA) protein assay (epizyme, China).

### Cell Proliferation Assay

For colony formation assay, Hela and C33a cells were seeded in 6‐well plates (5000 cells/well), and HL‐60 and SPP1/ELOVL5/GBP1 overexpression HL‐60 cells were cultured in upper chambers in transwell plates. After 1‐2 weeks of co‐culture, colonies were stained with crystal violet (Beyotime, C0121).

### Migration and Invasion Assay

After being co‐cultured with HL‐60 and SPP1/GBP1/ELOVL5 overexpression HL‐60 cells for 3 days, Hela and C33a cells cells were loaded in the upper chamber of transwell plates or the wells of a Matrigel plate (8µm pore size; Corning), and incubated at 37 °C for 36–48 h. Migrated cells were stained with crystal violet, and counted in 3 random high power fields.

### Metabonomic Analysis

Liquid chromatography and mass spectrometry liquid chromatography spectrometry tandem mass was performed according to the: previously developed method.^[^ ^36^ ^]^ HL‐60 cells and ELOVL5^oe^ HL‐60 cells were washed with pre‐cooled PBS. A total of 1 × 10^7^ of cells are rapidly frozen in liquid nitrogen for 10 min. Fatty acid metabolites were detected by MetWare (http://www.metware.cn/) based on the Agilent 8890‐5977B GC‐MS platform.

### Tube Formation Assay

The pipet tips and 12‐well plates were precooled, and growth‐factor‐reduced Matrigel (BD, Corning, USA) was thawed overnight prior to the assay. The wells of the 12‐well plate were coated with 200 µl of Matrigel and incubated for 2 h in 4 °C. After being co‐cultured with SPP1^oe^ HL‐60/HL‐60 cells for 2 days, huvecs were starved in ECM without serum for 24 h prior to the assay. Collected huvecs 24 h later and implanted them into 12‐wells plate with matrixgel, with 2 × 10^4^ cells each well. The tube formation of HUVECs was observed at 12h experimental period using a microscope.

### Statistical Analysis

Continuous variables are reported as medians with interquartile ranges (IQRs) or means with standard deviations (SDs). Comparisons of continuous data of different data types between two groups were performed using the unpaired two‐tailed Student t‐ test; one‐way analysis of variance with the Tukey post hoc test and Two‐way ANOVA analysis of variance with Bonferroni post hoc tests was used to compare data between multiple groups. Kaplan‐Meier curves between the two groups were analyzed using the log‐rank test. All statistical analyses were performed with GraphPad Prism (version 8, GraphPad Software) or R (version 4.1.2). Statistical significance was set at *p* < 0.05.

## Conflict of Interest

The authors declare no conflict of interest.

## Author Contributions

X.C., X.Q., and T.R. contributed equally to this work. The research study was designed by J.Q., K.H., and X.C. Data were collected by X.C., X.Q., and T.R., while T.W. and J.D. performed the data analysis. The manuscript was prepared by X.C. and T.X., and sample collection was carried out by T.R. and X.Q. Grammar checking was conducted by J.Q. and X.C. The study was supervised by J.D., K.H., and X.Q., and funding was acquired by K.H. and J.Q. The authors read and approved the final manuscript.

## Supporting information



Supporting Information

## Data Availability

The data that support the findings of this study are available on request from the corresponding author. The data are not publicly available due to privacy or ethical restrictions.
