# Supplementary material for: Decoding Triphenotypic Neutrophils in Cervical Cancer Evolution and Targeting SPP1+/GBP1+/ELOVL5+ Tumor‐Associated Neutrophils to Sensitize Immunotherapy
Source: Adv Sci (Weinh). 2025 Nov 28;13(29):e15511. doi: 10.1002/advs.202515511 (PMC13205737; doi:10.1002/advs.202515511)
Supplement: Supplementary file 1 — Supporting Information [file ADVS-13-e15511-s001.pdf]

Supplementary information

Neutrophils: evolving from defenders and agitators to promoters, highlighting the prominence of tumour-associated neutrophils for enhancing immunotherapeutic efficacy in cervical cancer

Table S1 Patient sample information

Clinical and pathological features					
	Sample1	Sample2	Sample3	Sample4	Sample5
HPV infection	Yes	Yes	Yes	Yes	Yes
Postoperative stage	IIa1	IIa1	IIIa	IIIa	IIIa
Maximum tumor diameter	2.5cm	3cm	4cm	3.9cm	3.5cm

Table S2 : Clinical characteristics of each clinical sample and numbers of cells sequenced after quality control procedures

Sample	Pathological Diagnosis	High-risk HPV Infection	Sequencing Method	Number of cells
HPV-N1	Cervical polyp	Negative	scRNA-seq	11619
HPV-N2	Normal cervix	Negative	scRNA-seq	9203
HPV+N1	Normal cervix	Positive	scRNA-seq	16218
HPV+N2	Chronic cervicitis	Positive	scRNA-seq	7335
HSIL	HSIL	Positive	scRNA-seq	6346
HSIL	HSIL	Positive	scRNA-seq	7371
SCC1	squamous cell carcinoma	Positive	scRNA-seq	3428
SCC2	squamous cell carcinoma	Positive	scRNA-seq	6914
SCC3	squamous cell carcinoma	Positive	scRNA-seq	5970
SCC4	squamous cell carcinoma	Positive	scRNA-seq	8979
SCC5	squamous cell carcinoma	Positive	scRNA-seq	6734
SCC6	adenocarcinoma of cervix, usual type	Positive	scRNA-seq	5753
SCC7	adenocarcinoma of cervix, usual type	Positive	scRNA-seq	2049

Table S3: Antibodies used for flow cytometry analysis.

Reagents and antibodies

Type	Source	Cat No.	Name
Reagents	Bioxcell	BE0075-1	In vivoMAb anti-mouse Ly6G antibody
	Bioxcell	BE0089	In vivoMAb rat lgG2a isotype control, anti-trinitrophenol antibody
	Bioxcell	BE0146	In vivoMAb anti-mouse PD-1 (CD279) antibody
Antibodies	Abcam	ab109361	Anti-ICAM1
	Abcam	ab32536	Anti-NF-kB p65
	Abclonal	ab307406	Anti-ITGB2
	Abcam	ab205921	Anti-PD-L1
	Abcam	ab108393	Anti-CD74
	Abcam	ab254183	Anti-CD11c
	Invitrogen	ab238145	Anti-LILRB1
	Affinity	ab126624-10ul	Anti-HLAF
	Affinity	ab238132	Anti-Ly6g
	Abcam	ab52627	Anti-Notch1
	Abcam	ab52895	Anti-CD11a
	Abcam	ab214050	Anti-Osteopontin
	proteintech	26455-1-AP	LILRB1 Polyclonal
	affinity	DF4038	Anti-ELOVL5
	Abcam	ab52895	Anti-CD11a
	Abcam	ab192238	Anti-SRPK2
	Abcam	ab305301	Anti-IFIT1
	Abcam	ab45690	Anti-CCL4/MIP-1 beta
	Abcam	ab131255	Anti-GBP1
	Abcam	ab186731	Anti-MIA
	Abcam	ab205535	Anti-ELOVL5
	Abcam	ab300122	Anti-CD66b
	Abcam	ab46154	Anti-VEGFA
	proteintech	55451-1-AP	Anti-CLEC9A Polyclonal
	BD Pharmingen	562899	BV421 Mouse Anti-Ki-67
	BD Pharmingen	563299	BV605 Rat Anti-Mouse Ly-6G
	Biolegend	124313	PE/Cy7 anti-mouse PD-L1
	BD Pharmingen	560584	PerCP-Cy5.5 Hamster Anti-Mouse CD11c
	Biolegend	143906	APC anti-mouse CD63
	Biolegend	143503	PE anti-mouse CLEC9A
	Proteintech	10268-1-AP	Anti-IkB Alpha
	Proteintech	15649-1-AP	Anti-IKBKB
	Abcam	ab51608	Anti-HIF-1 alpha

Figure S1

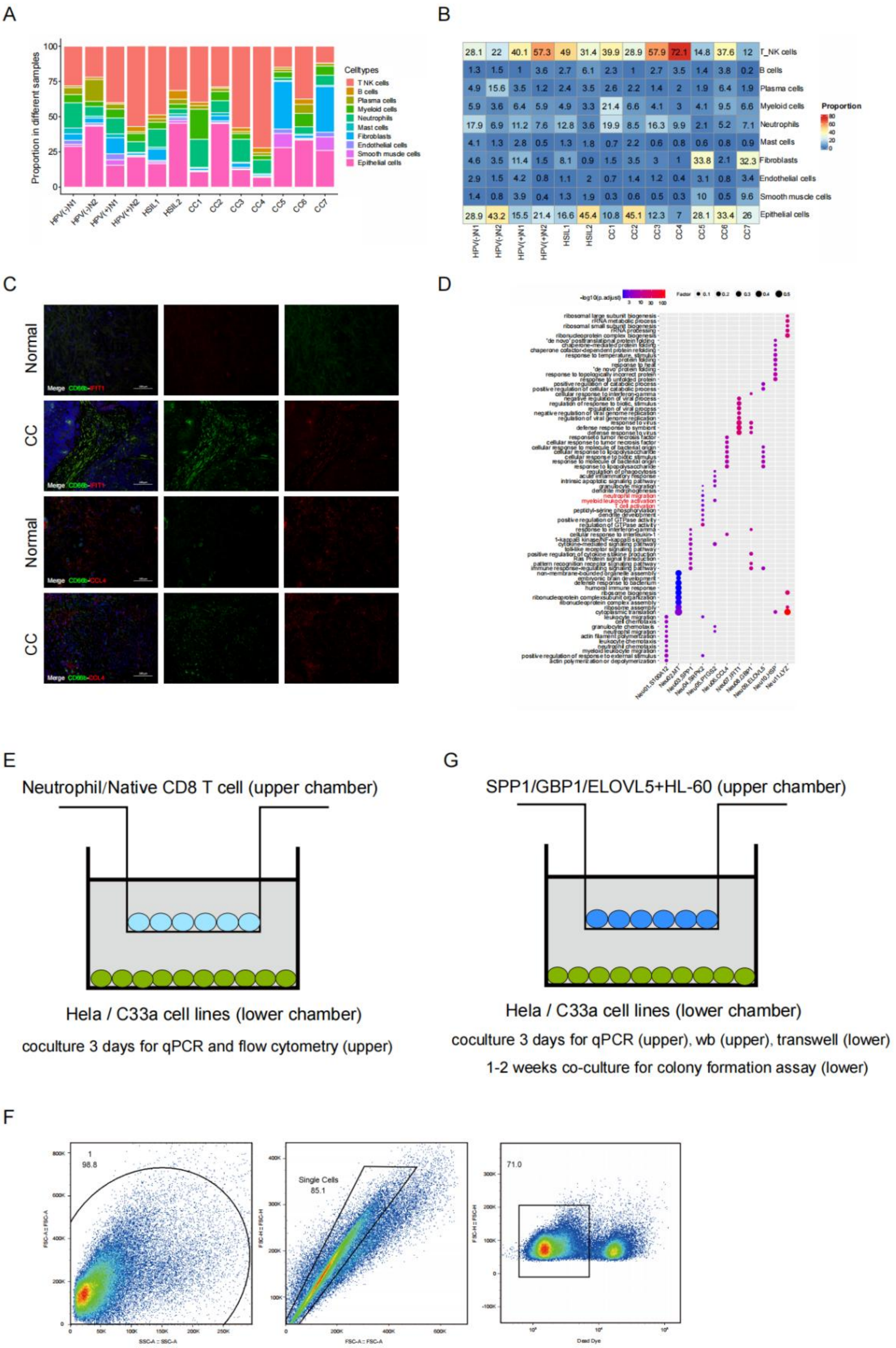
A
Proportion in different samples
Celltypes
T NK cells
B cells
Plasma cells
Myeloid cells
Neutrophils
Mast cells
Fibroblasts
Endothelial cells
Smooth muscle cells
Epithelial cells
HPV(-)N1
HPV(-)N2
HPV(+)N1
HPV(+)N2
HSIL1
HSIL2
CC1
CC2
CC3
CC4
CC5
CC6
CC7
B
Proportion
T_NK cells
B cells
Plasma cells
Myeloid cells
Neutrophils
Mast cells
Fibroblasts
Endothelial cells
Smooth muscle cells
Epithelial cells
C
Normal
CC
Merge CD66b IFIT1
Merge CD66b CCL4
D
E
Neutrophil/Native CD8 T cell (upper chamber)
Hela / C33a cell lines (lower chamber)
coculture 3 days for qPCR and flow cytometry (upper)
G
SPP1/GBP1/ELOVL5+HL-60 (upper chamber)
Hela / C33a cell lines (lower chamber)
coculture 3 days for qPCR (upper), wb (upper), transwell (lower)
1-2 weeks co-culture for colony formation assay (lower)
F
Single Cells
85.1
98.8
71.0
SSC-A :: SSC-A
FSC-A :: FSC-A
Dead Dye

Figure S1

A-B: Cell proportions of assigned cell types across different phases in 13 samples.

C: Representative multicolour immunofluorescence images showing the colocalisation of Neu06.CCL4 cells (CCL4+) and Neu07.IFIT1 cells (IFIT1+) in normal cervical tissue samples and CC tissue samples. Scale bars, 100 μm.

D: Analysis of GO functional enrichment of different neutrophil cluster.

E: Schematic illustration of Neutrophils/Native CD8 T cells-Hela/C33a cell lines co-culture system.

F：Flow cytometry of neutrophil phylum diagram.

G: Schematic illustration of SPP1/GBP1/ELOVL5 HL-60 cells-Hela/C33a cell lines co-culture system.

GO analysis: Gene Ontology analysis.

Figure S2

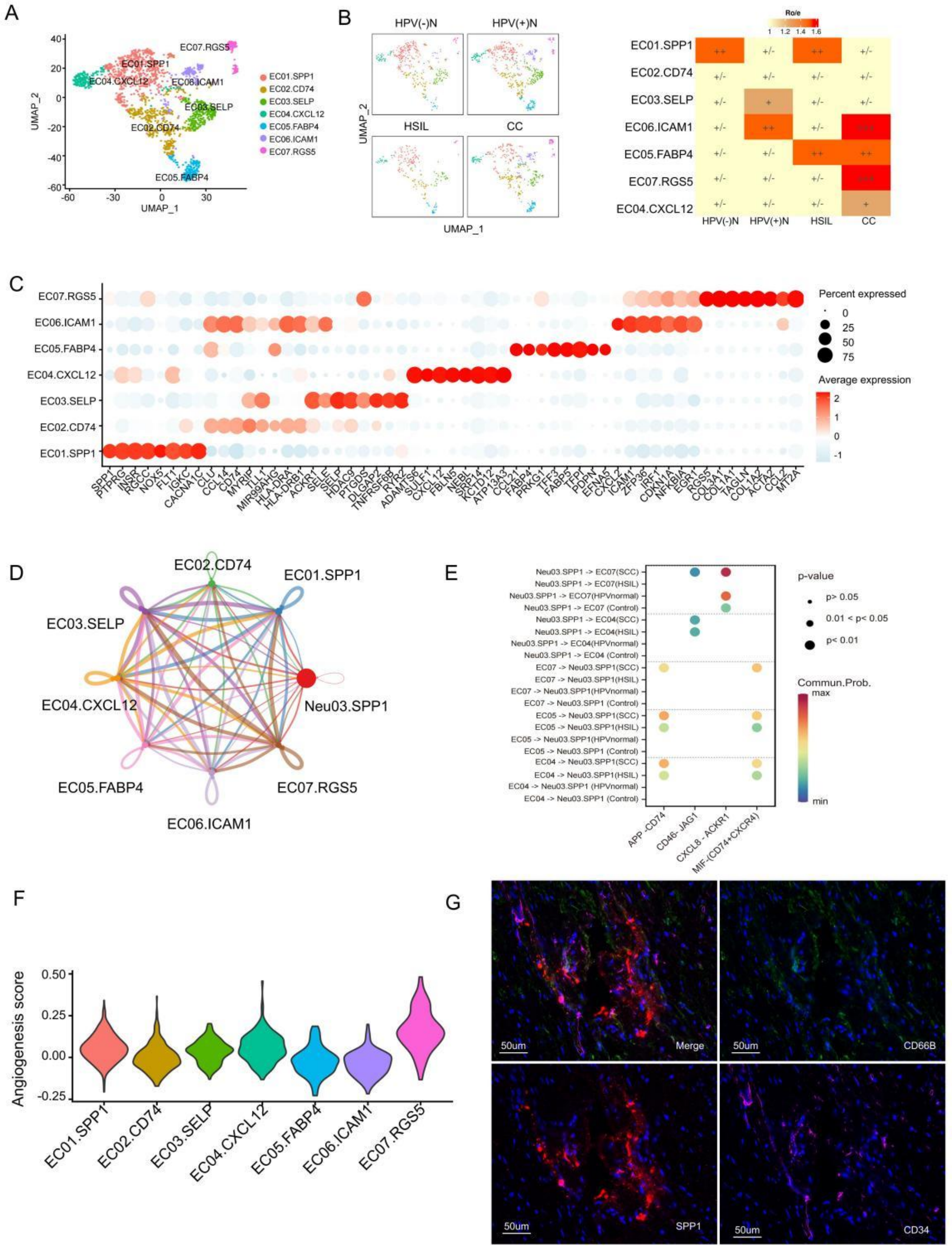

A
B
C
D
E
F
G
EC01.SPP1
EC02.CD74
EC03.SELP
EC04.CXCL12
EC05.FABP4
EC06.ICAM1
EC07.RGS5
UMAP_1
UMAP_2
HPV(-)N
HPV(+)N
HSIL
CC
Ro/e
Percent expressed
Average expression
Neu03.SPP1
p-value
p> 0.05
0.01 < p< 0.05
p< 0.01
Commun.Prob.
max
min
APP -CD74
CD46 - JAG1
CXCL8 - ACKR1
MIF-(CD74+CXCR4)
Angiogenesis score
Merge
CD66B
SPP1
CD34
50um

Figure S2: Single-cell transcriptome of endothelial cells from 13 samples.

A: The UMAP plot showing 7 EC clusters.

B: UMAP projection of different EC clusters in different tissue origins (Left); Tissue preference of EC clusters in humans, revealed by Ro/e (Right, ratio of observed cell number to expected cell number). EC04.CXCL12 and EC07.RGS5 clusters were mainly enriched in CC samples and designated tumour-associated ECs.

C: Expression of cell type-specific marker genes in different EC clusters.

D: Crosstalk between Neu03.SPP1 and EC subtypes. The width of the edges indicates the relative strength of the interactions.

E: Cell–cell connections show the specific legend-receptor pairs between $SPP1^{+}$TAN, $CXCL12^{+}$EC and $RGS5^{+}$EC subclusters.

F: Angiogenesis score of EC clusters.

G: Three-colour overlay images validated the colocalisation of SPP1+TANs (CD66b+, SPP1+) and microvascular (CD34+) through mIHC with CD66b (green), SPP1 (red), CD34 (pink). Scale bars, 50 μm.

Figure S3

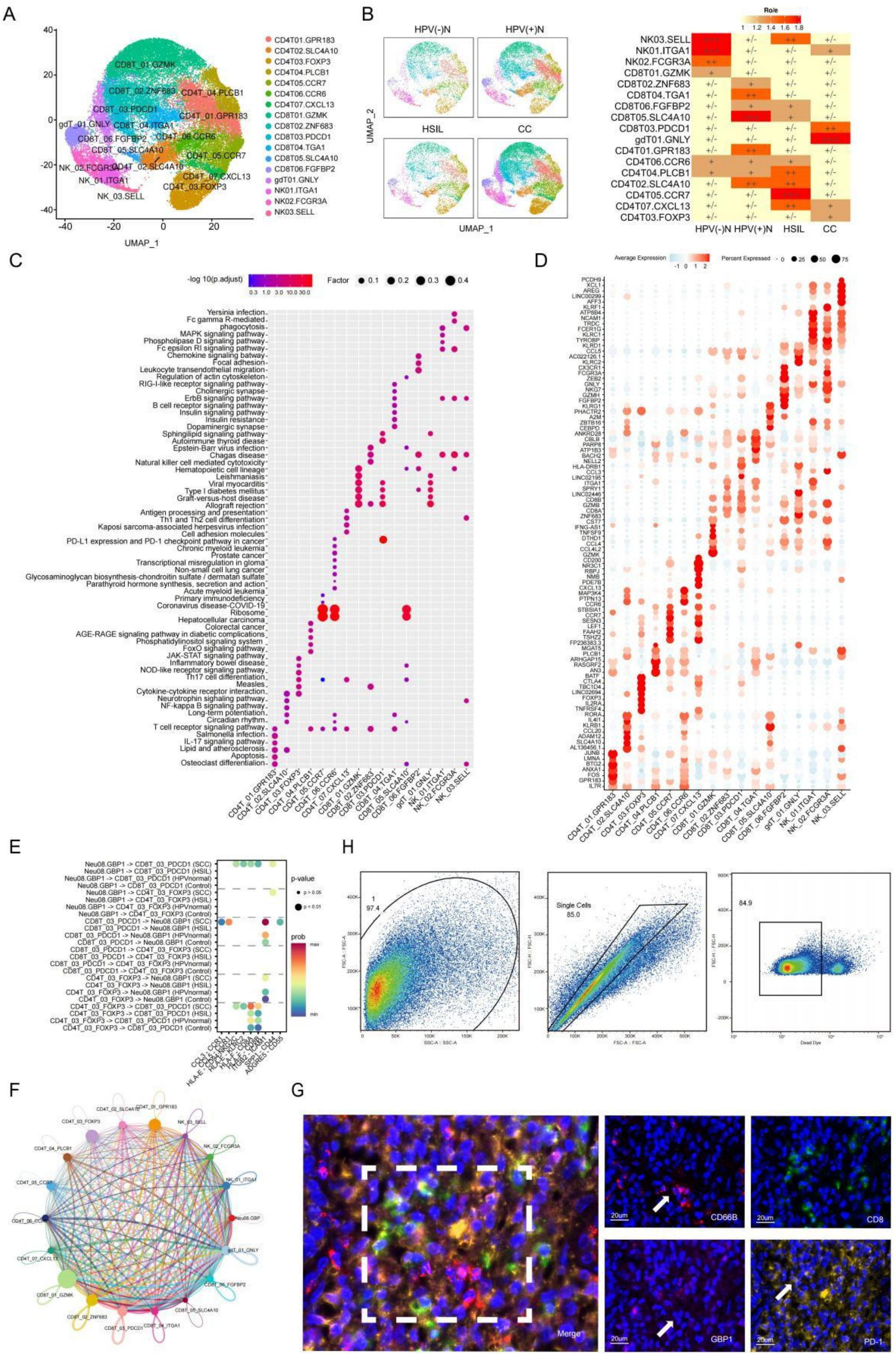

A
B
C
D
E
F
G
H
UMAP_1
UMAP_2
CD4T01.GPR183
CD4T02.SLC4A10
CD4T03.FOXP3
CD4T04.PLCB1
CD4T05.CCR7
CD4T06.CCR6
CD4T07.CXCL13
CD8T01.GZMK
CD8T02.ZNF683
CD8T03.PDCD1
CD8T04.TGA1
CD8T05.SLC4A10
CD8T06.FGFBP2
gdT01.GNLY
NK01.ITGA1
NK02.FCGR3A
NK03.SELL
HPV(-)N
HPV(+)N
HSIL
CC
Ro/e
-log 10(p.adjust)
Factor
Average Expression
Percent Expressed
p-value
prob
Single Cells
85.0
97.4
84.9
Dead Dye
CD66B
CD8
GBP1
PD-1
Merge
20um

Figure S3: Single-cell transcriptome of NKT cells from 13 samples.

A: The UMAP plot showing 17 NKT cell clusters.

B: UMAP projection of different NKT clusters in different tissue origins (Left); Tissue preference of EC clusters in humans, revealed by Ro/e (Right, ratio of observed cell number to expected cell number).

C: Analysis of KEGG functional enrichment of different NKT cell clusters.

D: Expression of cell type-specific marker genes in different NKT cell clusters.

E: Cell–cell connections show the specific legend-receptor pairs between GBP1+TAN, $PDCD1^{+}CD8$ T cell and $FOXP3^{+}CD4$ T cell subclusters. Based on the annotation results, PDCD1+CD8+ T cells were exhausted T cells (Tex) and FOXP3+CD4+ T cells as regulatory T cells (Tregs).

F: Crosstalk between Neu08.GBP1 and NKT subtypes. The width of the edges indicates the relative strength of the interactions.

G：Four-colour overlay images validated the colocalisation of GBP1+TANs (CD66b+, GBP1+) and PD-1+CD8 T cell (PD-1+CD8) through mIHC with CD66b (red), GBP1 (pink), CD8 (green) and PD-1 (yellow). Scale bars, 20 μm.

H：Flow cytometry of CD8 T cell phylum diagram.

KEGG: Kyoto Encyclopedia of Genes and Genomes.
